# Ibogaine and addiction in the animal model, a systematic review and meta-analysis

**DOI:** 10.1038/tp.2016.71

**Published:** 2016-05-31

**Authors:** M Belgers, M Leenaars, J R Homberg, M Ritskes-Hoitinga, A F A Schellekens, C R Hooijmans

**Affiliations:** 1 IrisZorg, Department of Addiction Health Care, Arnhem, The Netherlands; 2 Department of Psychiatry, Radboud University Medical Center, Nijmegen, The Netherlands; 3 Nijmegen Institute for Scientist-Practitioners in Addiction (NISPA), Nijmegen, The Netherlands; 4 Departments of SYstematic Review Centre for Laboratory animal Experimentation (SYRCLE), Central Animal Laboratory, Radboud University Medical Center, Nijmegen, The Netherlands; 5 Behavioural Neurogenetics group at the Department of Cognitive Neuroscience, Donders Institute for Brain, Cognition, and Behaviour, Radboud University Medical Center, Nijmegen, The Netherlands

## Abstract

Ibogaine is a naturally occurring substance which has been increasingly used in the lay-scene to reduce craving and relapse in patients with substance use disorders (SUDs). Although human clinical trials on the safety and efficacy of ibogaine are lacking, animal studies do support the efficacy of ibogaine. In this systematic review and meta-analysis (MA), we summarise these animal findings, addressing three questions: (1) does ibogaine reduce addictive behaviour in animal models of SUDs?; (2) what are the toxic effects of ibogaine on motor functioning, cerebellum and heart rhythm?; (3) what are neuropharmacological working mechanisms of ibogaine treatment in animal models of SUDs? MA of 27 studies showed that ibogaine reduced drug self-administration, particularly during the first 24 h after administration. Ibogaine had no effect on drug-induced conditioned place preference. Ibogaine administration resulted in motor impairment in the first 24 h after supplementation, and cerebral cell loss even weeks after administration. Data on ibogaines effect on cardiac rhythm, as well as on its neuropharmacological working mechanisms are limited. Our results warrant further studies into the clinical efficacy of ibogaine in SUD patients in reducing craving and substance use, but close monitoring of the patients is recommended because of the possible toxic effects. In addition, more work is needed to unravel the neuropharmacological working mechanisms of ibogaine and to investigate its effects on heart rhythm.

## Introduction

Substance use disorders (SUDs) account for a large share of the total global burden of disease. Nearly 5% of all disability-adjusted life years and 4% of overall mortality appear to be attributed to SUDs.^1, 2, 3^ SUDs are often characterized by chronicity and frequent relapse. Despite treatment, 5-year relapse rates are as high as 70% for alcohol dependence, 78% for cocaine dependence and 97% for opioid dependence.^4, 5, 6^ Moreover, for opioid dependence, pharmacological treatment mainly consists of harm reduction strategies, using opioid substitution with opioid agonists^7, 8^ and for cocaine dependence no effective pharmacological treatment is available at all.^9^

As a consequence, new and more effective pharmacological treatment modalities are needed. Several new treatments have been investigated, with some more promising than others. One promising compound is ibogaine, a naturally occurring substance in an African shrub. This compound has been claimed to reduce craving and relapse rates in patients with SUDs.^10^ Indeed, case reports mention a reduction of withdrawal symptoms and relapse after a single dose of ibogaine with a sustainability of this effect of several months.^11^ Ibogaine has increasingly been used for this purpose over the last decades, mainly in a lay-scene.^12, 13^ However, human clinical trials on the safety and efficacy of ibogaine for patients with SUDs are lacking.

Various animal studies seem to support the claim that ibogaine could have anti-addictive effects. The use of even a single dose of ibogaine appears to be effective in a variety of well-validated animal models for SUDs.^10, 14^ Other animal studies describe neurobiological effects of ibogaine.^15, 16^ These findings fit well with current insight into the pathophysiology of SUDs and its pharmacological targets, assigning a dominant role to dysfunction in the brain dopamine, serotonin and stress systems in SUDs.^17, 18^ However, a major concern in the use of ibogaine is its potential cerebellar and cardiac toxicity, which has been described in both animal studies and human case reports.^19, 20, 21^

In order to create an overview of possible therapeutic and adverse effects, and further our understanding of the neuropharmacological working mechanism of ibogaine, we conducted a systematic review (SR) and meta-analysis (MA) of animal studies regarding this topic. We propose that SR and MA of animal studies will increase our insight into the possible therapeutic effects, toxicity and potential mechanism of action of ibogaine. In addition, the results of this review might guide the design of future clinical trials.^22^ Therefore, three research questions will be addressed: (1) Does ibogaine reduce addictive behaviour in animal models of SUDs?; (2) Does ibogaine supplementation to animals cause adverse toxic effects?; and (3) Does ibogaine influence addiction-related neurobiological response in animal models of SUDs?

## Materials and methods

The present review was based on published results of the therapeutic, toxic and neurobiological effects of ibogaine in animal studies. The inclusion criteria and methods of analysis were specified in advance and documented in a protocol and published on the SYRCLE website (https://www.radboudumc.nl/Research/Organisationofresearch/Departments/cdl/SYRCLE/Pages/Protocols.aspx).

For our first research question (does ibogaine reduce addictive behaviour in animal models of SUDs?) we focused on the two main behavioural paradigms to measure features of SUDs: the drug self-administration (SA) and drug-induced conditioned place preference (CPP) paradigms. The drug SA paradigm measures the reinforcing effects of drugs of abuse, and—depending on the schedule of reinforcement used—the pattern, as well as motivation of animals to self-administer or seek drugs of abuse. The CPP paradigm measures the rewarding value of drugs of abuse and the ability of the animals to link this to the context in which they experience the reward.^23^ For our second research question (does ibogaine supplementation to animals cause adverse toxic effects?), we focused on the effect of ibogaine on motor functioning, cerebellar cell loss and cardiac rhythm effects, since tremors, ataxia and cardiac fatalities (due to cardiac arrhythmias) are the most commonly reported toxic effects of ibogaine in human case reports.^20^ For our third research question (does ibogaine influence addiction-related neurobiological response in animal models of SUDs?), we focused on studies reporting on dopaminergic and serotonergic effects of ibogaine, since these neurotransmitters have been reported to play a pivotal role in SUDs and relapse.^24, 25^ In this context, we defined an ‘animal model of SUD’ with the animals having experienced the same drug chronically, which means for at least two times.

### Search strategy and selection of the papers

We identified published manuscripts regarding the effects of ibogaine in animal models for SUDs in Medline via the PubMed interface, Embase, Psychinfo, CINAHL and Web of Science, until 1 November 2014. To minimise the risk of overlooking any studies, we applied a broad search strategy using the following five search terms:^26, 27^ ‘Ibogaine’, ‘noribogaine’, ‘12-Methoxyibogamine’, ‘NIH-10567’ and ‘Endabuse’ (for the complete search strategy see Supplementary Table 1). Reference lists of the selected papers were screened by hand for additional relevant papers.

Experimental animal studies were included in the SR if they fulfilled one of the following criteria: (a) the study employs ibogaine in a drug SA and/or drug-induced CPP paradigm; (b) the study is about the effect of ibogaine on cerebellar cell structure, motor functioning or cardiac rhythm; (c) the study is about the effect of ibogaine on dopaminergic or serotonergic neurotransmission in an animal with chronic drug use. Studies were excluded: (a) when they appeared to be a duplicate publication, review, letter or commentary; (b) when no control group was included in the experiment; (c) ibogaine treatment was combined with other drugs and (d) when the study was not an animal *in vivo* model.

Using Early Review Organizing Software (EROS; Institute of Clinical Effectiveness and Health Policy, Buenos Aires, Argentina), each reference was randomly allocated to two independent reviewers (MB and ML) who screened it for inclusion on the basis of title and abstract. In case of doubt, the whole publication was evaluated. Full-text copies of all publications eligible for inclusion were subsequently assessed by the same two reviewers and included if they met our pre-specified inclusion criteria. Disagreements were solved by discussion with a third investigator (CRH).

### Study characteristics and data extraction

From the included studies, bibliographic data such as authors, year of publication, journal of publication and language were registered. We also extracted data on study design (number of animals in the experimental and control groups), animal model characteristics (animal species, strain, age, body weight, and age at the beginning of the study and gender), intervention characteristics (description of addiction model, used drug in addiction model and its dosage regimen, ibogaine dosage regimen and administration route) and outcome measures. For our first research question (does ibogaine reduce addictive behaviour in animal models of SUDs?) outcome measures were SA of a drug (as quantised by number of active/inactive nose pokes, lever responses or drug infusions) or drug-induced CPP (as quantised by time spent in one compartment of a two-compartment chamber) of an animal. For our second research question (does ibogaine supplementation to animals cause adverse toxic effects?) outcome measures were occurrence and severity of motor impairment (including tremors and ataxia as measured by uphill orientation on, or falling off a tilted platform), cerebellar cell loss and cardiac toxicity. For our third research question (does ibogaine influence addiction-related neurobiological response in animal models of SUDs?) outcome measures were any proxies of dopamine and/or serotonin function in different brain regions.

If available, raw data or group averages (mean, median or incidence) were extracted, as well as s.d., s.e. or ranges and number of animals per group (*n*). If more than one experiment was reported on the same outcome measure in a manuscript, the experiments were only included separately in the analyses when other animals were used. Multiple experiments on the same group of animals were pooled. If the number of animals was reported as a range, the lowest number of animals was included in the analyses. If data were presented only graphically, we applied Universal Desktop Ruler software (http://avpsoft.com/products/udruler/), to come to an adequate estimation of the outcome measurements. In case of missing outcome measure data, we contacted the authors for additional information. If no adequate estimation could be made or data were missing, the results were excluded from the analyses. If multiple experimental groups were compared with the same control group, the group size of the control group was corrected for the number of comparisons made (*n*/number of comparisons).

### Assessment of methodological quality and risk of bias

The risk of bias was assessed for each of the included studies by two authors independently (MB and ML) using SYRCLE’s risk of bias tool.^28^ A ‘yes’ score indicates low risk of bias; a ‘no’ score indicates high risk of bias; and a ‘?’ score indicates unknown risk of bias. In case of disagreement, a third author was consulted (CRH). Concerning the number of excluded animals, we assumed that there had been no exclusion if the number of animals per group mentioned in the Materials and methods section was identical to the number stated in the Results section or figure legends. Reporting of experimental details on animals, methods and materials is often limited^29^ and to overcome the problem of judging too many items as ‘unclear risk of bias’ we added two items: reporting of any measure of randomisation; and reporting of any measure of blinding. For these two items, a ‘yes’ score indicates ‘reported’, and a ‘no’ score indicates ‘unreported’.

### Data synthesis and statistical analyses

MA was performed using Comprehensive Meta-Analysis software (version 2.2, Biostat, Englewood, NJ, USA). For all the continuous outcome measures, the s.d. was calculated if only the s.e. was reported (s.d.= s.e. × √*n*). In case, data were presented as median and percentiles, these data were converted to mean and s.d.^30^ In the data set on ibogaine toxicology, both continuous and dichotomous data were reported, which were analysed separately. When one of the cells to calculate a risk ratio (RR) contained a zero-value or the risk in either the control or experimental group was 100%, we added 0.5 to each cell to calculate the RR. Despite anticipated heterogeneity, individual effect sizes were pooled to obtain an overall standardized mean difference (SMD) for continuous outcome measures and a RR for dichotomous outcome measures, with their 95% confidence intervals. A random effects model^31^ was used, which takes the precision of individual studies and the variation between studies into account and weights each study accordingly.

Explorative subgroup analyses were performed for different dosages of ibogaine, different time frames for the effect, different animal species, gender and type of drug used, only if at least four studies were available per subgroup. These subgroups were specified in advance and documented in the protocol (www.syrcle.nl). Because dosing regimens varied considerably among studies, we also grouped studies into low, medium and high dose studies, corresponding with 0–40 mg kg^−1^, 40–80 mg kg^−1^ and >80 mg kg^−1^, respectively. Dosages used by humans are typically between 10 and 25 mg kg^−1^ orally.^12^ When translating these dosages to animal studies according body surface area^32^ and taking into account differences in effects after oral and i.p. dosing regimens^33^ they correspond with 40–80 mg kg^−1^. Because the starting point of measurements after the ibogaine supplementation varied considerably between studies, we grouped these in to three time frames, corresponding with 0–24 h, 24–72 h and >72 h after ibogaine supplementation, respectively. Since motor impairment is most often reported during the first 24 h after ibogaine administration, this could influence measurements. It is relevant to explore the effects beyond the acute phase of 24 h, since human reports claim ibogaine has a lasting effect for several months even after a single dose.^34^ Morphine and heroine (di-acetyl morphine) were grouped together under opioids since their working mechanism are almost identical. We assumed that the variance was comparable within the subgroups; therefore, we assumed a common among-study variance across subgroups. For subgroup analyses, we applied a Bonferroni correction for multiple testing (p* number of comparisons). However, differences between subgroups should be interpreted with caution and should only be used for constructing new hypotheses rather than for drawing final conclusions.

### Sensitivity analyses and publication bias analyses

We performed sensitivity analyses to assess the robustness of our findings, by changing the boundaries of dosage regimen and time frames. We also assessed whether analysing morphine and heroin separately would change the results for this group. Furthermore, we assessed the possibility of publication bias by evaluating symmetry in the funnel plot for the outcome measure SA, performing Duval and Tweedie's trim and fill analysis, and Egger's regression analysis for small study effects. Heterogeneity was assessed using *I*^2^.

## Results

### Study selection process and search results

**Figure 1:**
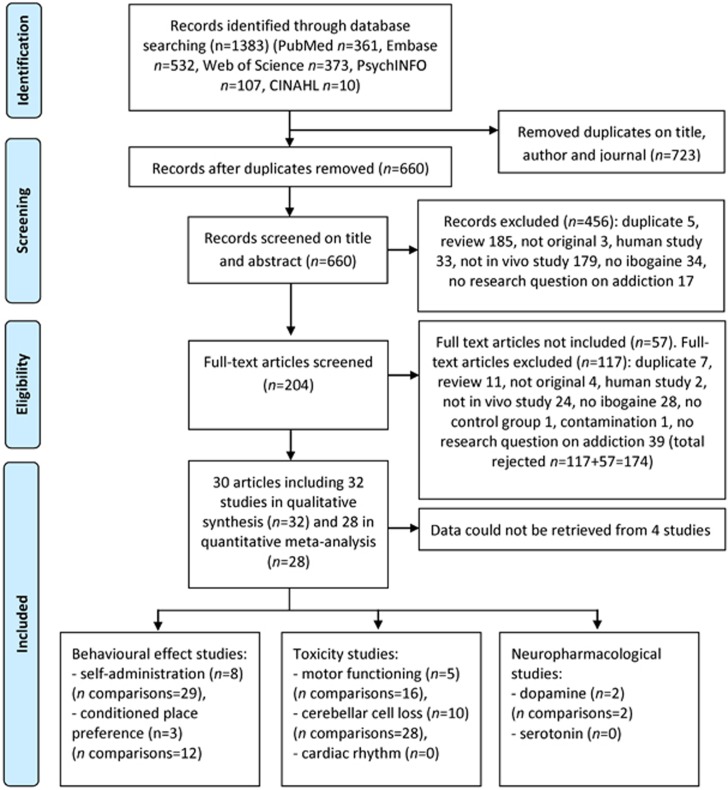
Flow chart of the study selection.

Our search yielded 361 records from PubMed, 532 articles from EMBASE, 107 from Psychinfo, 10 from CHINAHL and 373 of Web of Science. About 660 articles appeared to be unique (see Figure 1 for a consort flow chart). Ultimately, 30 articles (all in English language) matched the inclusion criteria (Supplementary Table 2).

These 30 articles contained 32 studies, which were included for SR (see Supplementary Table 3 for study characteristics). Eleven studies described the drug SA or CPP paradigms. Nineteen studies were on ibogaine toxicity. From four of these studies data could not be retrieved. There were no *in vivo* studies on the cardiac effects of ibogaine, which matched our inclusion criteria. Only two papers reported dopaminergic and/or serotonergic effects of ibogaine in animals that had chronic contact with drugs. The remaining 28 studies were analysed in MA. Most comparisons were conducted in rats (87%), and the remaining in mice. In the majority of comparisons, male animals were used (73%). Weight of the animals was reported in 21 studies (70%) and half of the studies reported the age of the animals. From the behavioural effect studies, four studies reported on the effect on cocaine use, four on morphine, one on heroin, one on amphetamine and one on alcohol use.

### Description of characteristics and MA of the effects of ibogaine in animals

#### Effect of ibogaine on drug SA

**Figure 2:**
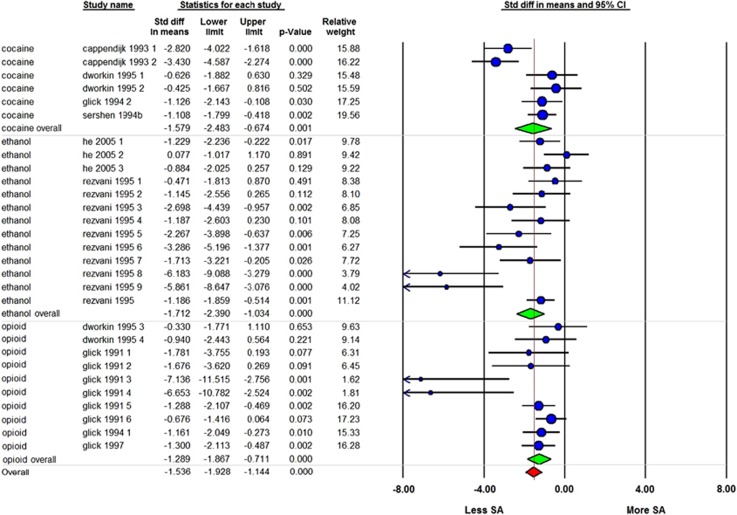
Forest plot of the impact of ibogaine on drug self-administration (SA). A negative outcome means reduction of SA; a positive outcome means an enhancement. Data are presented as standardized mean differences (SMD) and 95% confidence intervals (CI).

From 8 studies, 29 independent comparisons about the effect of ibogaine on drug SA could be included in MA. The data of 10 independent comparisons from 3 studies could not be retrieved for MA.^35, 36, 37^ MA indicates that ibogaine reduced drug SA (SMD=−1.54 [−1.93; −1.14] *n*=29; *I*^2^=64%), (Figure 2). In none of the comparisons, ibogaine enhanced drug SA.

**Figure 3:**
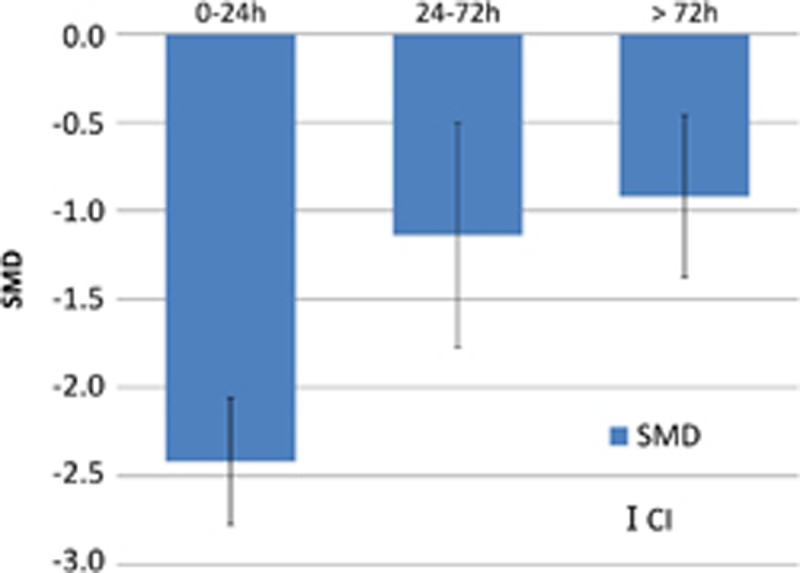
Effect (standard mean difference (SMD)) of ibogaine on drug self-administration (column, effect size; line, confidence interval (CI)) as function in three time frames (0–24 h, 24–72 h,>72 h) after ibogaine dosing.

Subgroup analyses revealed a larger reduction of SA in the first 24 h after ibogaine dosing (SMD=−2.42 [−3.05; −1.78] *n*=25; *I*^2^=71%), compared with measurements after this time point (24–78 h: SMD=−1.14 [−1.60;−0.68] *n*=13; *I*^2^=46% *P*=0.003, >72 h: SMD=−0.92 [−1.50; −0.34] *n*=9; *I*^2^=65% *P*=0.002) (Figure 3.).

There was no difference in the effect of ibogaine on SA between other subgroups, with different types of drugs being used (cocaine, opioids and ethanol), gender or dosing levels. There was also no difference between these subgroups if we analysed the prolonged effect of ibogaine on SA (measurements >24 h after ibogaine dosing) (Table 1). We could not analyse for species subgroup because the number of comparisons was <4. The overall in-between study heterogeneity was large (*I*^2^=64%).

#### Effect of ibogaine on drug-induced CPP

**Figure 4:**
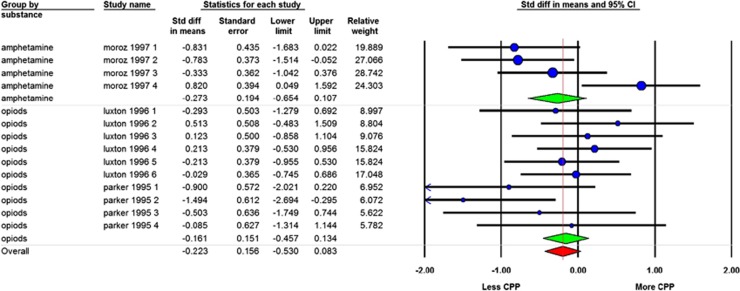
Forest plot of the impact of ibogaine on drug-induced conditioned place preference (CPP). A negative outcome means reduction of CPP; a positive outcome means an enhancement. Data are presented as standardized mean differences (SMD) and 95% coincidence intervals.

Fourteen independent comparisons from 3 studies reported about the effect of ibogaine on drug-induced CPP, 4 using amphetamine and 10 using morphine. Ibogaine did not reduce drug-induced CPP (SMD=−0.22 [−0.53; 0.08] *n*=14; *I*^2^=39% see Figure 4). Subgroup analyses were not performed for gender, strain and species, as all comparisons were done with male Sprague–Dawley rats. There were no differences in effects of ibogaine on drug-induced CPP in the comparison of subgroups, with different types of drugs being used (amphetamine and opioids), different time frames of measurement after ibogaine supplementation and dosing levels (Supplementary Table 5). There was also no difference between the subgroups of one cycle of CPP learning or two or more cycles. The overall in-between study heterogeneity was moderate (*I*^2^=39%).

#### Toxic effects of ibogaine

**Figure 5:**
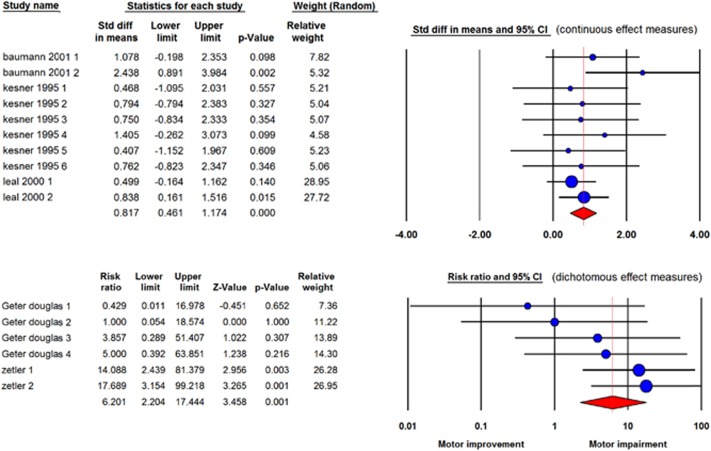
Forest plots for continuous (upper) and dichotomous (lower) effect measures of ibogaine on motor functioning. In the upper plot (continuous measurements), a negative outcome means enhancement of motor functioning; a positive outcome means an impairment. In this plot, data are presented as standardized mean differences (SMD) and 95% coincidence intervals. In the lower plot (dichotomous measurements), an outcome <1 means enhancement of motor functioning; an outcome >1 means an impairment of motor functioning. In this plot, data are presented as risk ratios and 95% coincidence intervals.

The effect of ibogaine on motor functioning: Eight studies reported on toxic effects of ibogaine on motor functioning, from which three study data could not be retrieved.^37, 38, 39^ From the 5 other studies, 10 independent comparisons with continuous outcome measures and 6 with dichotomous outcome measures reported about the effect of ibogaine on motor functioning. Both the continuous and dichotomous outcome measures showed that the administration of ibogaine caused motor impairment (continuous: SMD=0.82 [0.46; 1.17] *n*=10; *I*^2^=0%, dichotomous: RR=6.20 [2.20; 17.44] *n*=6; *I*^2^=15; see Figure 5). All measurements were obtained within 24 h after ibogaine administration. In all comparisons male animals were used. Eight comparisons used rats, and the other eight used mice.

There was no difference in the occurrence of motor symptoms after ibogaine between different dosing regimens (continuous measurements: <40 mg kg^−1^: SMD=1.11 [0.44; 1.78] *n*=5; *I*^2^=0% 40–80 mg kg^−1^: SMD=0.61 [0.07; 1.15] *n*=4 *I*^2^=0% dichotomous measurements: <40 mg kg^−1^: RR=5.61 [1,15; 17.44] *n*=4; *I*^2^=46% see Supplementary Table 6). However, subgroups on dosages of >80 mg kg^−1^ were too small to analyse, as well as the subgroup of medium dosage in the group of dichotomous measurements. The in-between study heterogeneity was low for both the continuous and dichotomous outcome measures (*I*^2^=0% and 15%, respectively).

**Figure 6:**
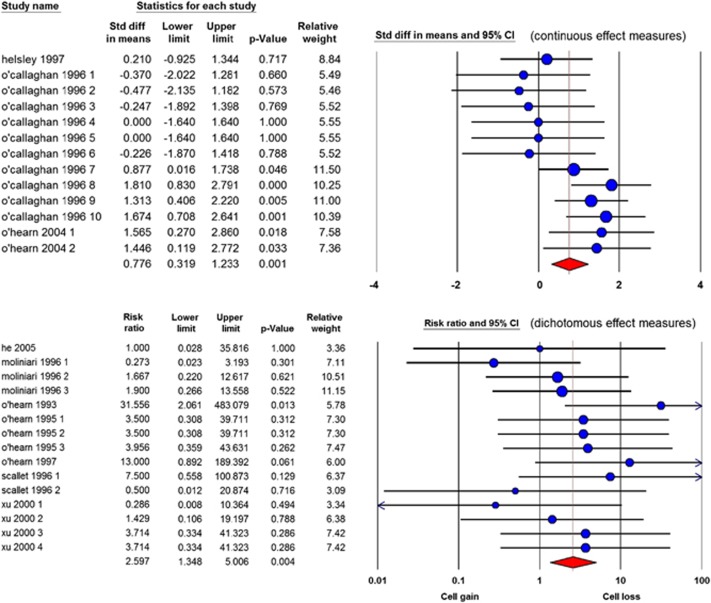
Forest plots for continuous (upper) and dichotomous (lower) effect measures of ibogaine on cerebellar cell loss. In the upper plot (continuous measurements), a negative outcome means reduction of cerebellar cell loss; a positive outcome means an enhancement. In this plot, data are presented as standardized mean differences (SMD) and 95% confidence intervals. In the lower plot (dichotomous measurements), an outcome <1 means that no cerebellar cell loss has occurred, an outcome >1 means cerebellar cell loss has occurred. In this plot, data are presented as risk ratios and 95% coincidence intervals.

The effect of ibogaine on cerebellar cell loss: Eleven studies reported on toxic effects of ibogaine on cerebellar cell loss. From the 11 studies, 1 study data could not be retrieved.^40^ From the 10 other, 28 comparisons reported on the effects of ibogaine administration on cerebellar degeneration. Indicators of cerebellar degeneration applied were the Nadler–Evenson method, glial fibrillary acidic protein immunocytochemical, fluoro-jade or anti-calbinidin staining or according to immune reactivity with antibodies against calcium-calmodulin-dependent protein kinase II (Cam KII). Both the continuous and dichotomous outcome measures showed that administration of ibogaine causes cerebellar cell loss (continuous: SMD=0.78 [0.32; 1.23] *n*=13; *I*^2^=42%, dichotomous: RR=2.60 [1.35; 5.01] *n*=15; *I*^2^=0; see Figure 6).

In the group with continuous measurements, six comparisons applied oral ibogaine administration. In the dichotomous group, ibogaine was administered i.p. The effect of ibogaine on cerebral cell loss was only observed in comparisons with i.p. administration, but not after oral administration (i.p.: SMD= 1.27 [0.87; 1.66] *n*=7; *I*^2^=4% oral: SMD=−0.22 [−0.89; 0.45] *n*=6 *I*^2^=0%).

There was no effect of gender on the results, nor was there a difference between mice and rats (analysed in the dichotomous studies). In the group of continuous measurements, all animals were rats, and in this group we could not analyse potential differences between dosing groups because the number of comparisons was <4. However, in the group with dichotomous measurements no effect was found in the medium dosing groups in contrast with the high dosing group (Supplementary Table 7). In the group with continuous measurements only, cell loss was observed when measured >72 h after ibogaine supplementation. The overall in-between study heterogeneity was moderate in the group with continuous measurements (*I*^2^=42%) and zero in the group with dichotomous measurements.

The effect of ibogaine on heart rhythm: No studies were found on cardiovascular effects of ibogaine that matched our inclusion criteria.

#### Neuropharmacological effects of ibogaine in animal models of SUDs

Only two studies matched our inclusion criteria.^15, 16^ These studies showed that ibogaine treatment lowered drug-induced dopamine efflux in rats, as measured with dialysate levels in the nucleus accumbens and striatum after chronic cocaine or morphine use (SMD=−1.14 [−2.03; −0,26] *n*=2; *I*^2^=0%).

#### Summary of results

See Table 2 for a summary of the results.

### Risk of bias, quality of reporting and publication bias

Of the 30 studies included in this SR, only few applied methods to avoid bias. Five studies (17%) reported randomisation of treatment in some way. However, none of these studies mentioned the methods of randomisation applied. None of the papers stated that the experiments were blinded, described the allocation sequence or concealing during the randomisation process. Measures to reduce performance bias (random housing and blinding of the caregivers) were reported in only one study. None of the studies reported that the outcome assessor was blinded for the allocation of the animals. Only one study reported random outcome assessment. In 27% of the studies, the baseline characteristics varied between the control group and experimental group at the start of the experiment. Most potential sources of bias had to be scored as unclear risk of bias, as a consequence of poor reporting (Supplementary Figure 1).

Inspection of the funnel plots suggested some asymmetry due to an underrepresentation of studies with moderate to low precision and increased drug SA after ibogaine use. Duval and Tweedie's trimm and fill analysis for the data set on drug SA resulted in 4 extra data points of the total of 29 comparisons, indicating slight overestimation of the summary effect size (Supplementary Figure 2). Sensitivity analysis revealed that changing the boundaries of our inclusion criteria and the classification of our subgroups did not alter our results significantly.

## Discussion

The current MA on the effect of ibogaine treatment in animal models of SUDs is, to the best of our knowledge, the first of its kind. Ibogaine was found to reduce SA of cocaine, ethanol and opioids in animals, but lacked an effect on CPP learning paradigms. The effect on SA lasted for over 72 h after ibogaine administration, indicating potentially long-term beneficial effects. Ibogaine treatment also induced motor impairment during the first 24 h after ibogaine administration, independent of the dosage used. Dose-dependent cerebellar cell loss was observed even weeks after ibogaine administration. No animal studies regarding cardiac toxicity of ibogaine treatment were identified. Similarly, studies on the neuropharmacology of ibogaine treatment in animal models of SUDs are limited as well.

The observation that ibogaine reduces SA of cocaine, ethanol and opioids is consistent with clinical observations in humans and previous narrative reviews of animal studies.^10, 12, 14, 41^ Though the beneficial effects of ibogaine were most prominent in the first 24 h after administration, the beneficial effects lasted for over 72 h. This is in line with human case reports, describing long-lasting effects of ibogaine on craving in patients with cocaine and opiate dependence, for several months.^42, 43^

While ibogaine decreases drug SA, it is not effective in reducing drug-induced CPP. One difference between the two paradigms is that CPP measures aspects of Pavlovian conditioning of an animal experiencing rewarding or salient effects of a substance in a given context. Pavlovian conditioning is an automatic, involuntarily reflex-like process. The drug SA paradigm, on the other hand, involves a combination of Pavlovian and operant conditioning. A cue presented in the test cage signals the availability of the substance through Pavlovian conditioning, serving as a conditioned reinforcer. In addition, based on both the conditioned reinforcer and the rewarding effects of the substance itself the animals are reinforced to press on a lever or to poke into a nose-poke-hole to obtain the substance. Hence, active behaviour is required in the drug SA paradigm, which may eventually develop as a habit. Because of the voluntarily nature of the drug SA paradigm, this paradigm has greater translational value compared with the CPP paradigm. It is tempting to speculate that ibogaine reduces drug SA, reflecting an effect on the reinforcing properties of a substance as measured by operant conditioning, but not Pavlovian conditioning that is linked to the rewarding effects of drugs.

We found that ibogaine causes acute motor impairment in animals, even in low dosages. No studies published results on whether these effects last over time, although three of the five studies described that tremors and ataxia disappeared within 24 h.^44, 45, 46^ This would be consistent with human studies, where these effects also disappeared within 24 h.^47^ It is hypothesised that these effects on motor functioning are caused by ibogaines’ exciting effect on neurons in the inferior olivary nucleus, within the medulla oblongata. Sustained release of glutamate in neurons of the olivary nucleus triggered by ibogaine may contribute to excitotoxic degeneration of cells within the cerebellum.^48^ Indeed, high doses of ibogaine were associated with reduced cerebellar cell counts. It has been suggested that the motor effects of ibogaine mediate the beneficial effects of ibogaine on drug SA. However, some studies report an absence of the effect of ibogaine on water-intake, making this hypothesis less likely.^49^

We did not encounter animal studies focussing on the effects of ibogaine on the heart rhythm, matching our inclusion criteria. However, we found one study showing a decrease in heart rate, blood pressure and cardiac output in four anaesthetised dogs receiving 5 mg kg^−1^ of ibogaine besides barbital.^50^ We also found some animal studies about the cardiovascular effect of tabernanthine (an isomer of ibogaine, different in the position of a methoxyl group) reporting bradycardia and hypotension.^51, 52, 53^ Mash *et al.*^47^ reported that no electrocardiographic abnormalities were seen in 150 patients treated with ibogaine. Notably, in this study it is unclear which exact methods were used for monitoring. Other human studies do report potentially life-threatening effects of ibogaine on heart rhythm.^21^ Some *in vitro* studies report an effect of ibogaine on the human Ether-à-go-go-Related Gene potassium channel, potentially contributing to prolonged QT intervals on the electrocardiogram and eventually cardiac arrhythmias.^54^ This is particularly relevant, since SUD patients often use other QT prolonging medication (like methadone and SSRI’s). Moreover, studies reported bradycardia, hypotension and a diminished cardiac output after use of an ibogaine-isomer, which the authors attributed to an effect on calcium mobilisation.^50, 51^ Since cardiac effects of ibogaine could be related to previously reported fatalities after ibogaine ingestion^20^ and intoxications,^55^ there is a great need for animal studies addressing this issue.

Although we conducted a very comprehensive search on the neuropharmacology of ibogaines’ effects in animal models of addiction, only two articles matched our inclusion criteria. This clearly points out that there is a gap of knowledge regarding the neuropharmacological mechanisms of action of ibogaine in addiction. Nevertheless, these two studies showed that ibogaine treatment lowered drug-induced dopamine efflux in rats chronically exposed to drugs. Consistent with the theoretical role of dopamine in addiction, this could explain why ibogaine is capable of reducing drug SA in animals and why it may reduce craving and relapse in patients with SUDs.

Because of the well-established role of specifically dopaminergic and serotonergic signalling in drug craving, risk-related decision making, impulse control and relapse in addiction research,^56, 57^ we decided to explore the pharmacological effects of ibogaine on these pathways only. However, several other neurotransmitter systems are relevant in the context of addiction, particularly for alcohol given its effects on a variety of brain receptors. Given the limited number of studies identified in our search, we conducted retrospective analyses on our search results to identify studies that investigated (1) other neuropharmacological effects of ibogaine and (2) other animal models of substance use, including single dose studies.

The first retrospective analysis identified one extra article on cerebral glucose utilisation, indicating ibogaine reduces cerebral glucose utilisation in morphine-dependent rats.^58^ This paper did not report on any effect on specific neurotransmitter systems. The second retrospective analysis identified 14 additional articles (summarised in Supplementary Table 4). In these articles, 11 studies were about the effect of ibogaine on drug-induced dopamine efflux. Different drugs of abuse were used and measurements focused on different brain areas. Five studies showed ibogaine reduced this drug-induced efflux of dopamine, three studies showed an enhancement and three showed no effect. This lack of a clear direction of effect when animals received a drug only once is in contrast with our finding of ibogaines positive effect in reducing dopamine efflux in the animal forced with more than one drug-suppletion. This could be due to the limited amount of studies we found, but it would also be consistent with our findings that ibogaine affects drug-induced reinforcement learning (as seen in drug SA) but not Pavlovian conditioning (as seen in drug-induced CPP).

Studies on the acute effects of ibogaine in animals not exposed to any drug of abuse show that ibogaine can block *N*-Methyl-d-aspartate-type glutamatergic neurotransmission^59^ and inhibit glial glutamate re-uptake.^60^ Other potential neuropharmacological mechanisms of ibogaine proposed in the literature include antagonism on the α3β4 nicotinic acetylcholine receptor ^61^ and serotonin transporter,^62^ effects on gene expression including some transcription factors involved in SUDs (like Fos family protein (ΔFosB) and cAMP response element binding protein)^63^ and the enhancement of glial cell line-derived neurotrophic factor.^64, 65^ However, the exact neuropharmacological mechanism of ibogaine in the treatment of SUDs remains to be elucidated.

## Strengths and limitations of the review

The findings of this MA should be seen in the light of its strengths and limitations. A particularly strong point is the systematic and meta-analytic approach to summarise all available animal evidence regarding the effects of ibogaine for addiction. Such an evidence-based approach maximises the changes of successful translation from bench to bedside.^66^

However, there are also some limitations to our study. First, the risk of bias analysis showed poor reporting in most studies concerning essential methodological details. As a consequence most items in the risk of bias tool for animal studies^28^ had to be scored as unclear risk of bias. It is unclear whether this insufficiency in reporting truly reflects hampered methodological rigour, contributing to bias, confounding or skewed findings.^67^ Nevertheless, for both scientific and ethical reasons methodological reporting of individual animal studies urgently needs to be improved.^22, 68, 69^

Second, this SR/MA contains a relatively low number of studies and revealed generally moderate to large levels of heterogeneity. This combination may diminish the certainty in the effect estimates. Nevertheless, to account for the observed heterogeneity we used a random (rather than fixed) effects model in the MA and conducted subgroup analyses. In addition, one needs to bear in mind that animal studies are often explorative and heterogeneous with respect to species, design and intervention protocols and so on, as compared with clinical trials, and moderate levels of heterogeneity might therefore be expected.

Third, several limitations of the SA and the CPP models of SUDs should be taken into account. Though the SA model is the best behavioural paradigm for predicting medication effects for the treatment of cocaine and opioid dependence,^70^ and is increasingly used in animal studies on SUDs,^71^ treatments which are effective in SA and CPP do not always translate to humans. For example, not all aspects of SUDs in humans, like socioeconomics, peer group, and co-morbidity can be modelled in animal models. In addition, we did not find studies on the effect of ibogaine on SA of other drugs of abuse than opioids, cocaine and ethanol. Therefore, the current findings with ibogaine cannot be generalised to all SUDs. Yet, studies with 18-Methoxycoronaridine (a synthetic analogue of ibogaine) and noribogaine (a metabolite of ibogaine) did also show reduced SA for amphetamine and nicotine.^65, 72, 73, 74, 75, 76^

Finally, no studies took phenotypic animal variation into account, although this may be highly relevant in the context of SUDs. For example, some rats are more attracted to conditioned stimuli predicting reward rather than to the reward (goal) itself. These so-called sign-trackers are more motivated to self-administer cocaine,^77^ and are highly dependent on dopamine. Dopamine release increases on presentation of a conditioned stimulus predicting reward in these sign-trackers.^78^ Since ibogaine blocks dopamine release in the NAc,^15^ it is tempting to speculate that sign-trackers are more responsive to ibogaine than goal-trackers (attracted to reward delivery). Similarly, trait anxiety and impulsivity differentially contribute to the liability to SA and CPP,^79^ and could influence the efficacy of ibogaine.

## Recommendations and clinical relevance

Our SR clearly indicates that ibogaine has potentially strong and long-lasting reducing effects on the amount of drug SA in animals. However, administration of ibogaine to animals also causes negative effects, as impaired motor function and cerebellar cell loss in high dosages given i.p. are observed. In addition, although we did not find clear evidence regarding cardiac toxic effects of ibogaine, clinical experiences with ibogaine do suggest that ibogaine can cause potentially fatal cardiac arrhythmias. Given the great clinical need for effective treatment modalities for addiction, translational studies on the effects of ibogaine on drug craving and relapse in humans are urgently needed. To minimise risks of heart arrhythmias and cerebellar cell loss, we recommend oral and low dosages of ibogaine to be applied in human studies, only under close medical monitoring, and after thorough medical screening.

Last but not least, with this SR, we identified several gaps of knowledge in the preclinical literature on the effects of ibogaine. Animal studies should further unravel the neuropharmacological mechanisms mediating the effects of ibogaine on substance use. Further, there is an urgent need for improving methodological reporting of animal studies, to improve the successful translation of animal research outcomes into the human clinical setting.

## Figures and Tables

**Table 1:**
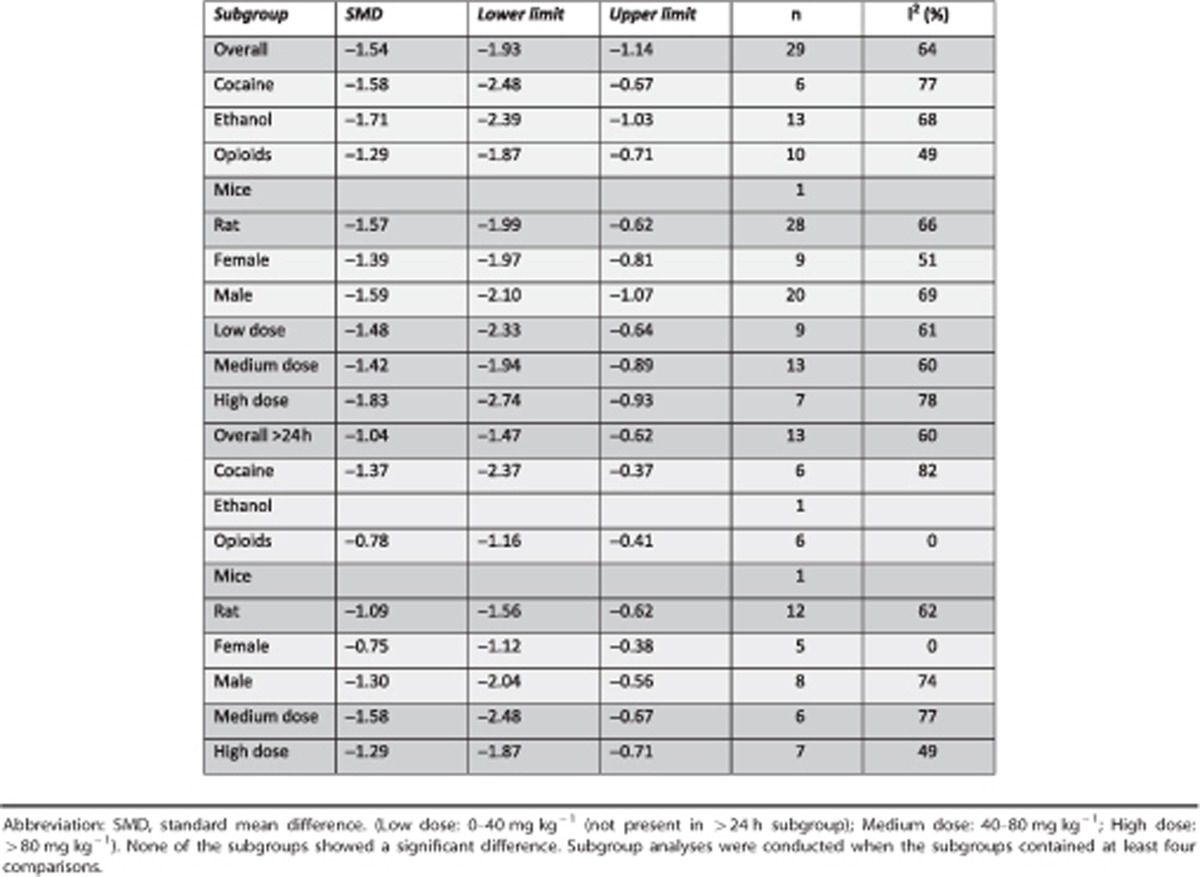
Effects of ibogaine on drug self-administration in different subgroups for all comparisons (blue, *n*=29) and for all comparisons 24 h after ibogaine dosing (green, *n*=13)

**Table 2:**
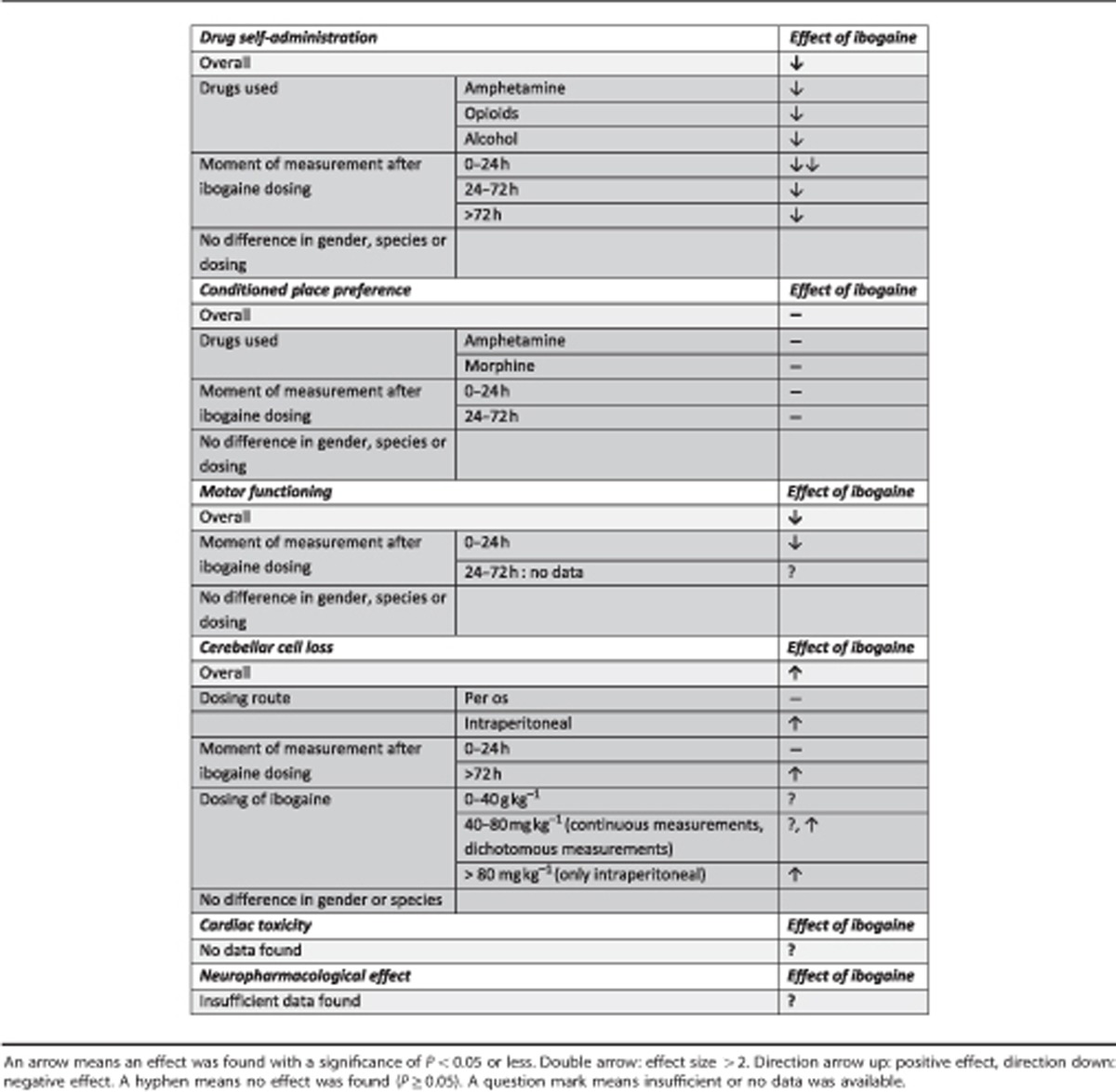
Summary of results

## References

[bib1] Rehm J. Global alcohol-attributable deaths from cancer, liver cirrhosis, and injury in 2010. Alcohol Res 2014; 35: 174–183. 10.35946/arcr.v35.2.07 PMC3908708 24881325

[bib2] Whiteford HA, Degenhardt L, Rehm J, Baxter AJ, Ferrari AJ, Erskine HE et al. Global burden of disease attributable to mental and substance use disorders: findings from the Global Burden of Disease Study 2010. Lancet 2013; 382: 1575–1586. 23993280 10.1016/S0140-6736(13)61611-6

[bib3] Wittchen HU, Jacobi F, Rehm J, Gustavsson A, Svensson M, Jonsson B et al. The size and burden of mental disorders and other disorders of the brain in Europe 2010. Eur Neuropsychopharmacol 2011; 21: 655–679. 21896369 10.1016/j.euroneuro.2011.07.018

[bib4] Duvall HJ, Locke BZ, Brill L. Followup study of narcotic drug addicts five years after hospitalization. Public Health Rep 1963; 78: 185–194. 19316439 PMC1915236

[bib5] Finney JW, Hahn AC, Moos RH. The effectiveness of inpatient and outpatient treatment for alcohol abuse: the need to focus on mediators and moderators of setting effects. Addiction 1996; 91: 1773–1796, discussion 1803-1720. 8997760

[bib6] Schoenthaler SJ, Blum K, Braverman ER, Giordano J, Thompson B, Oscar-Berman M et al. NIDA-Drug Addiction Treatment Outcome Study (DATOS) relapse as a function of spirituality/religiosity. J Reward Defic Syndr 2015; 1: 36–45. 26052556 10.17756/jrds.2015-007 PMC4455957

[bib7] Jerry JM, Collins GB. Medication-assisted treatment of opiate dependence is gaining favor. Clev Clin J Med 2013; 80: 345–349. 10.3949/ccjm.80a.12181 23733899

[bib8] Larney S, Gowing L, Mattick RP, Farrell M, Hall W, Degenhardt L. A systematic review and meta-analysis of naltrexone implants for the treatment of opioid dependence. Drug Alcohol Rev 2014; 33: 115–128. 24299657 10.1111/dar.12095

[bib9] Shorter D, Domingo CB, Kosten TR. Emerging drugs for the treatment of cocaine use disorder: a review of neurobiological targets and pharmacotherapy. Exp Opin Emerg Drugs 2015; 20: 15–29. 10.1517/14728214.2015.985203 25425416

[bib10] Brown TK. Ibogaine in the treatment of substance dependence. Curr Drug Abuse Rev 2013; 6: 3–16. 23627782 10.2174/15672050113109990001

[bib11] Alper KR, Lotsof HS, Frenken GM, Luciano DJ, Bastiaans J. Treatment of acute opioid withdrawal with ibogaine. Am J Addict 1999; 8: 234–242. 10506904 10.1080/105504999305848

[bib12] Alper KR, Lotsof HS, Kaplan CD. The ibogaine medical subculture. J Ethnopharmacol 2008; 115: 9–24. 18029124 10.1016/j.jep.2007.08.034

[bib13] Alper KR, Lotsof HS. The use of ibogaine in the treatment of addictions. In: Winkelman M, Roberts TB (eds). Psychedelic medicine: new evidence for hallucinogenic substances as treatments. Vol 2. Praeger Perspectives: Westport, CT, 2007, pp 43–66.

[bib14] Maciulaitis R, Kontrimaviciute V, Bressolle FM, Briedis V. Ibogaine, an anti-addictive drug: pharmacology and time to go further in development. A narrative review. Hum Exp Toxicol 2008; 27: 181–194. 18650249 10.1177/0960327107087802

[bib15] Szumlinski KK, Maisonneuve IM, Glick SD. Differential effects of ibogaine on behavioural and dopamine sensitization to cocaine. Eur J Pharmacol 2000; 398: 259–262. 10854838 10.1016/s0014-2999(00)00325-3

[bib16] Pearl SM, Maisonneuve IM, Glick SD. Prior morphine exposure enhances ibogaine antagonism of morphine-induced dopamine release in rats. Neuropharmacology 1996; 35: 1779–1784. 9076757 10.1016/s0028-3908(96)00116-5

[bib17] Volkow ND, Baler RD. Addiction science: uncovering neurobiological complexity. Neuropharmacology 2014; 76 Pt B: 235–249. 23688927 10.1016/j.neuropharm.2013.05.007 PMC3818510

[bib18] Glick SD, Maisonneuve IM, Szumlinski KK. Mechanisms of action of ibogaine: relevance to putative therapeutic effects and development of a safer iboga alkaloid congener. Alkaloid Chem Biol 2001; 56: 39–53. 10.1016/s0099-9598(01)56006-x 11705115

[bib19] Xu Z, Chang LW, Slikker W Jr, Ali SF, Rountree RL, Scallet AC. A dose-response study of ibogaine-induced neuropathology in the rat cerebellum. Toxicol Sci 2000; 57: 95–101. 10966515 10.1093/toxsci/57.1.95

[bib20] Alper KR, Stajic M, Gill JR. Fatalities temporally associated with the ingestion of ibogaine. J Forensic Sci 2012; 57: 398–412. 22268458 10.1111/j.1556-4029.2011.02008.x

[bib21] Koenig X, Hilber K. The anti-addiction drug ibogaine and the heart: a delicate relation. Molecules 2015; 20: 2208–2228. 25642835 10.3390/molecules20022208 PMC4382526

[bib22] Wever KE, Menting TP, Rovers M, van der Vliet JA, Rongen GA, Masereeuw R et al. Ischemic preconditioning in the animal kidney, a systematic review and meta-analysis. PLoS One 2012; 7: e32296. 22389693 10.1371/journal.pone.0032296 PMC3289650

[bib23] George O, Koob GF. Individual differences in prefrontal cortex function and the transition from drug use to drug dependence. Neurosci Biobehav Rev 2010; 35: 232–247. 20493211 10.1016/j.neubiorev.2010.05.002 PMC2955797

[bib24] Budygin EA, Weiner JL. Exploring the neurochemical basis of alcohol addiction-related behaviors: translational research. Transl Biomed 2015; 6(Suppl Spec): pii; PMID: 26770883. PMC4710378 26770883

[bib25] Müller CP, Homberg JR. The role of serotonin in drug use and addiction. Behav Brain Res 2015; 277: 146–192. 24769172 10.1016/j.bbr.2014.04.007

[bib26] de Vries RB, Hooijmans CR, Tillema A, Leenaars M, Ritskes-Hoitinga M. Updated version of the Embase search filter for animal studies. Lab Anim 2014; 48: 88. 23836850 10.1177/0023677213494374

[bib27] Hooijmans CR, Tillema A, Leenaars M, Ritskes-Hoitinga M. Enhancing search efficiency by means of a search filter for finding all studies on animal experimentation in PubMed. Lab Anim 2010; 44: 170–175. 20551243 10.1258/la.2010.009117 PMC3104815

[bib28] Hooijmans CR, Rovers MM, de Vries RB, Leenaars M, Ritskes-Hoitinga M, Langendam MW. SYRCLE's risk of bias tool for animal studies. BMC Med Res Methodol 2014; 14: 43. 24667063 10.1186/1471-2288-14-43 PMC4230647

[bib29] Kilkenny C, Parsons N, Kadyszewski E, Festing MF, Cuthill IC, Fry D et al. Survey of the quality of experimental design, statistical analysis and reporting of research using animals. PLoS One 2009; 4: e7824. 19956596 10.1371/journal.pone.0007824 PMC2779358

[bib30] Hozo SP, Djulbegovic B, Hozo I. Estimating the mean and variance from the median, range, and the size of a sample. BMC Med Res Methodol 2005; 5: 13. 15840177 10.1186/1471-2288-5-13 PMC1097734

[bib31] DerSimonian R, Laird N. Meta-analysis in clinical trials. Control Clin Trials 1986; 7: 177–188. 3802833 10.1016/0197-2456(86)90046-2

[bib32] Reagan-Shaw S, Nihal M, Ahmad N. Dose translation from animal to human studies revisited. FASEB J 2008; 22: 659–661. 17942826 10.1096/fj.07-9574LSF

[bib33] Kubiliene A, Marksiene R, Kazlauskas S, Sadauskiene I, Razukas A, Ivanov L. Acute toxicity of ibogaine and noribogaine. Medicina (Kaunas) 2008; 44: 984–988. 19142057

[bib34] Sheppard SG. A preliminary investigation of ibogaine: case reports and recommendations for further study. J Subst Abuse Treat 1994; 11: 379–385. 7966509 10.1016/0740-5472(94)90049-3

[bib35] Cappendijk SL, Dzoljic MR. Inhibitory effects of ibogaine on cocaine self-administration in rats. Eur J Pharmacol 1993; 241: 261–265. 8243561 10.1016/0014-2999(93)90212-z

[bib36] Dworkin SI, Gleeson S, Meloni D, Koves TR, Martin TJ. Effects of ibogaine on responding maintained by food, cocaine and heroin reinforcement in rats. Psychopharmacology 1995; 117: 257–261. 7770600 10.1007/BF02246099

[bib37] Glick SD, Kuehne ME, Raucci J, Wilson TE, Larson D, Keller RW Jr et al. Effects of iboga alkaloids on morphine and cocaine self-administration in rats: relationship to tremorigenic effects and to effects on dopamine release in nucleus accumbens and striatum. Brain Res 1994; 657: 14–22. 7820611 10.1016/0006-8993(94)90948-2

[bib38] Chen K, Kokate TG, Donevan SD, Carroll FI, Rogawski MA. Ibogaine block of the NMDA receptor: *In vitro* and *in vivo* studies. Neuropharmacology 1996; 35: 423–431. 8793904 10.1016/0028-3908(96)84107-4

[bib39] Trouvin JH, Jacqmin P, Rouch C, Lesne M, Jacquot C. Benzodiazepine receptors are involved in tabernanthine-induced tremor: *in vitro* and *in vivo* evidence. Eur J Pharmacol 1987; 140: 303–309. 2820763 10.1016/0014-2999(87)90287-1

[bib40] O'Hearn E, Molliver ME. Degeneration of Purkinje cells in parasagittal zones of the cerebellar vermis after treatment with ibogaine or harmaline. Neuroscience 1993; 55: 303–310. 8377927 10.1016/0306-4522(93)90500-f

[bib41] Alper KR. Ibogaine: a review. Alkaloid Chem Biol 2001; 56: 1–38. 10.1016/s0099-9598(01)56005-8 11705103

[bib42] Mash DC, Kovera CA, Pablo J, Tyndale RF, Ervin FD, Williams IC et al. Ibogaine: complex pharmacokinetics, concerns for safety, and preliminary efficacy measures. Ann N Y Acad Sci 2000; 914: 394–401. 11085338 10.1111/j.1749-6632.2000.tb05213.x

[bib43] Schenberg EE, de Castro Comis MA, Chaves BR, da Silveira DX. Treating drug dependence with the aid of ibogaine: a retrospective study. J Psychopharmacol 2014; 28: 993–1000. 25271214 10.1177/0269881114552713

[bib44] Baumann MH, Rothman RB, Pablo JP, Mash DC. *In vivo* neurobiological effects of ibogaine and its O-desmethyl metabolite, 12-hydroxyibogamine (noribogaine), in rats. J Pharmacol Exp Ther 2001; 297: 531–539. 11303040

[bib45] Kesner RP, Jackson-Smith P, Henry C, Amann K. Effects of ibogaine on sensory-motor function, activity, and spatial learning in rats. Pharmacol Biochem Behav 1995; 51: 103–109. 7617719 10.1016/0091-3057(94)00367-r

[bib46] Leal MB, de Souza DO, Elisabetsky E. Long-lasting ibogaine protection against NMDA-induced convulsions in mice. Neurochem Res 2000; 25: 1083–1087. 11055745 10.1023/a:1007665911622

[bib47] Mash DC, Kovera CA, Pablo J, Tyndale R, Ervin FR, Kamlet JD et al. Ibogaine in the treatment of heroin withdrawal. Alkaloid Chem Biol 2001; 56: 155–171. 10.1016/s0099-9598(01)56012-5 11705106

[bib48] O'Hearn E, Molliver ME. The olivocerebellar projection mediates ibogaine-induced degeneration of Purkinje cells: a model of indirect, trans-synaptic excitotoxicity. J Neurosci 1997; 17: 8828–8841. 9348351 10.1523/JNEUROSCI.17-22-08828.1997 PMC6573067

[bib49] Glick SD, Pearl SM, Cai J, Maisonneuve IM. Ibogaine-like effects of noribogaine in rats. Brain Res 1996; 713: 294–297. 8725004 10.1016/0006-8993(95)01563-9

[bib50] Schneider JA, Rinehart RK. Analysis of the cardiovascular action of ibogaine hydrochloride. Arch Int Pharmacodyn Ther 1957; 110: 92–102. 13425751

[bib51] Hamon G, Castillon A, Gaignault JC, Worcel M. Peripheral cardiovascular effects of tabernanthine tartrate in anaesthetized rats. Arch Int Pharmacodyn Ther 1985; 276: 60–72. 4051640

[bib52] Zetler G, Lenschow E, Prenger-Berninghoff W. ?Die wirkung von 11 indol alkaloiden auf das Meerschweinchenherz *in vivo* und *in vitro*, verglichen met 2 synthetischen Azepinoindolen, chinidin und quindonium. Naunyn Schmiedebergs Arch Pharmakol Exp Pathol 1968; 260: 226–249. 4236373

[bib53] Hajo N, Dupont C, Wepierre J. [Effects of tabernanthine on various cardiovascular parameters in the rat and dog (author's transl)]. J Pharmacol 1981; 12: 441–453. 7321573

[bib54] Koenig X, Kovar M, Boehm S, Sandtner W, Hilber K. Anti-addiction drug ibogaine inhibits hERG channels: a cardiac arrhythmia risk. Addict Biol 2014; 19: 237–239. 22458604 10.1111/j.1369-1600.2012.00447.x PMC4888945

[bib55] Vlaanderen L, Martial LC, Franssen EJ, van der Voort PH, Oosterwerff E, Somsen GA. Cardiac arrest after ibogaine ingestion. Clin Toxicol 2014; 52: 642–643. 10.3109/15563650.2014.927477 24940646

[bib56] Orsini CA, Moorman DE, Young JW, Setlow B, Floresco SB. Neural mechanisms regulating different forms of risk-related decision-making: Insights from animal models. Neurosci Biobehav Rev 2015; 58: 147–167. 26072028 10.1016/j.neubiorev.2015.04.009 PMC7913606

[bib57] Garbusow M, Sebold M, Beck A, Heinz A. Too difficult to stop: mechanisms facilitating relapse in alcohol dependence. Neuropsychobiology 2014; 70: 103–110. 25359490 10.1159/000362838

[bib58] Levant B, Pazdernik TL. Differential effects of ibogaine on local cerebral glucose utilization in drug-naive and morphine-dependent rats. Brain Res 2004; 1003: 159–167. 15019575 10.1016/j.brainres.2003.12.032

[bib59] Skolnick P. Ibogaine as a glutamate antagonist: relevance to its putative antiaddictive properties. Alkaloid Chem Biol 2001; 56: 55–62. 10.1016/s0099-9598(01)56007-1 11705116

[bib60] Leal MB, Emanuelli T, Porciuncula LD, Souza DO, Elisabetsky E. Ibogaine alters synaptosomal and glial glutamate release and uptake. Neuroreport 2001; 12: 263–267. 11209932 10.1097/00001756-200102120-00017

[bib61] Glick SD, Maisonneuve IM, Kitchen BA, Fleck MW. Antagonism of alpha 3 beta 4 nicotinic receptors as a strategy to reduce opioid and stimulant self-administration. Eur J Pharmacol 2002; 438: 99–105. 11906717 10.1016/s0014-2999(02)01284-0

[bib62] Bulling S, Schicker K, Zhang YW, Steinkellner T, Stockner T, Gruber CW et al. The mechanistic basis for noncompetitive ibogaine inhibition of serotonin and dopamine transporters. J Biol Chem 2012; 287: 18524–18534. 22451652 10.1074/jbc.M112.343681 PMC3365767

[bib63] Nestler EJ. Transcriptional mechanisms of drug addiction. Clin Psychopharmacol Neurosci 2012; 10: 136–143. 23430970 10.9758/cpn.2012.10.3.136 PMC3569166

[bib64] He DY, McGough NN, Ravindranathan A, Jeanblanc J, Logrip ML, Phamluong K et al. Glial cell line-derived neurotrophic factor mediates the desirable actions of the anti-addiction drug ibogaine against alcohol consumption. J Neurosci 2005; 25: 619–628. 15659598 10.1523/JNEUROSCI.3959-04.2005 PMC1193648

[bib65] Carnicella S, He DY, Yowell QV, Glick SD, Ron D. Noribogaine, but not 18-MC, exhibits similar actions as ibogaine on GDNF expression and ethanol self-administration. Addict Biol 2010; 15: 424–433. 21040239 10.1111/j.1369-1600.2010.00251.x PMC3783954

[bib66] Lau J, Ioannidis JP, Schmid CH. Quantitative synthesis in systematic reviews. Ann Int Med 1997; 127: 820–826. 9382404 10.7326/0003-4819-127-9-199711010-00008

[bib67] Hirst JA, Howick J, Aronson JK, Roberts N, Perera R, Koshiaris C et al. The need for randomization in animal trials: an overview of systematic reviews. PLoS One 2014; 9: e98856. 24906117 10.1371/journal.pone.0098856 PMC4048216

[bib68] Hooijmans CR, de Vries RB, Rovers MM, Gooszen HG, Ritskes-Hoitinga M. The effects of probiotic supplementation on experimental acute pancreatitis: a systematic review and meta-analysis. PLoS One 2012; 7: e48811. 23152810 10.1371/journal.pone.0048811 PMC3496732

[bib69] Macleod MR, van der Worp HB, Sena ES, Howells DW, Dirnagl U, Donnan GA. Evidence for the efficacy of NXY-059 in experimental focal cerebral ischaemia is confounded by study quality. Stroke 2008; 39: 2824–2829. 18635842 10.1161/STROKEAHA.108.515957

[bib70] Haney M, Spealman R. Controversies in translational research: drug self-administration. Psychopharmacology 2008; 199: 403–419. 18283437 10.1007/s00213-008-1079-x PMC2731701

[bib71] Bossert JM, Marchant NJ, Calu DJ, Shaham Y. The reinstatement model of drug relapse: recent neurobiological findings, emerging research topics, and translational research. Psychopharmacology 2013; 229: 453–476. 23685858 10.1007/s00213-013-3120-y PMC3770775

[bib72] Glick SD, Maisonneuve IM, Dickinson HA. 18-MC reduces methamphetamine and nicotine self-administration in rats. Neuroreport 2000; 11: 2013–2015. 10884062 10.1097/00001756-200006260-00041

[bib73] Polston JE, Pritchett CE, Sell EM, Glick SD. 18-Methoxycoronaridine blocks context-induced reinstatement following cocaine self-administration in rats. Pharmacol Biochem Behav 2012; 103: 83–94. 22885280 10.1016/j.pbb.2012.07.013 PMC3526685

[bib74] Glick SD, Sell EM, McCallum SE, Maisonneuve IM. Brain regions mediating alpha3beta4 nicotinic antagonist effects of 18-MC on nicotine self-administration. Eur J Pharmacol 2011; 669: 71–75. 21871879 10.1016/j.ejphar.2011.08.001 PMC3183297

[bib75] Maisonneuve IM, Glick SD. Anti-addictive actions of an iboga alkaloid congener: a novel mechanism for a novel treatment. Pharmacol Biochem Behav 2003; 75: 607–618. 12895678 10.1016/s0091-3057(03)00119-9

[bib76] King CH, Meckler H, Herr RJ, Trova MP, Glick SD, Maisonneuve IM. Synthesis of enantiomerically pure (+)- and (-)-18-methoxycoronaridine hydrochloride and their preliminary assessment as anti-addictive agents. Bioorg Med Chem Lett 2000; 10: 473–476. 10743951 10.1016/s0960-894x(00)00033-0

[bib77] Saunders BT, Robinson TE. Individual variation in the motivational properties of cocaine. Neuropsychopharmacology 2011; 36: 1668–1676. 21471956 10.1038/npp.2011.48 PMC3138662

[bib78] Flagel SB, Clark JJ, Robinson TE, Mayo L, Czuj A, Willuhn I et al. A selective role for dopamine in stimulus-reward learning. Nature 2011; 469: 53–57. 21150898 10.1038/nature09588 PMC3058375

[bib79] Homberg JR, Karel P, Verheij MM. Individual differences in cocaine addiction: maladaptive behavioural traits. Addict Biol 2014; 19: 517–528. 24835358 10.1111/adb.12036

