# Supplementary material for: Ibogaine and addiction in the animal model, a systematic review and meta-analysis
Source: Transl Psychiatry. 2016 May 31;6(5):e826–. doi: 10.1038/tp.2016.71 (PMC5545647; doi:10.1038/tp.2016.71)
Supplement: Supplementary Information [file tp201671x1.doc]

**Supplement**

***Table 1***. Search Strategy

| PubMed | Component 1: "Ibogaine"[Mesh] OR ibogaine[tiab] OR noribogaine[tiab] OR 12-Methoxyibogamine[tiab] OR 12 Methoxyibogamine[tiab] OR NIH-10567[tiab] OR NIH 10567[tiab] OR Endabuse[tiab]  Component 2: search filter for animal studies [^1^](#_ENREF_1) |
| --- | --- |
| Embase | Component 1: (exp ibogaine/OR (ibogaine or ibogain or noribogain or nor-ibogaine or nor-ibogain or noribogaine or NIH-10567 or "NIH 10567" or NIH10567 or Endabuse).ti,ab.))  *12-Methogyibogamine and 12 Methogyibogamine gives a syntax error in search string*  Component 2: search filter for animal studies |
| PsychINFO | Component 1: (ibogaine or ibogain or noribogain or nor-ibogaine or nor-ibogain or noribogaine or NIH-10567 or NIH10567 or Endabuse).ti,ab.  *12-Methogyibogamine and 12 Methogyibogamine gives a syntax error in search string*  Component 2: search filter for animal studies |
| CINAHL | Component 1: TI ( ibogaine OR ibogain OR noribogain OR nor-ibogaine OR nor-ibogain OR noribogaine OR 12-Methoxyibogamine OR “12 Methoxyibogamine” OR NIH-10567 OR “NIH 10567” OR NIH10567 OR Endabuse ) OR AB ( ibogaine OR ibogain OR noribogain OR nor-ibogaine OR nor-ibogain OR noribogaine OR 12-Methoxyibogamine OR “12 Methoxyibogamine” OR NIH-10567 OR “NIH 10567” OR NIH10567 OR Endabuse  Component 2: search filter for animal studies |
| Web of Science | Component 1: Topic=(ibogaine OR ibogain OR noribogain OR nor-ibogaine OR nor-ibogain OR noribogaine OR 12-Methoxyibogamine OR “12 Methoxyibogamine” OR NIH-10567 OR “NIH 10567” OR NIH10567 OR Endabuse)  *Databases=SCI-EXPANDED, SSCI, A&HCI Timespan=All Years; Lemmatization=On*  Component 2: search filter for animal studies |

***Table 2****. Reference list of the included articles*

| Baumann | 2001 | [^4^](#_ENREF_4) Baumann MH, Rothman RB, Pablo JP, Mash DC. In vivo neurobiological effects of ibogaine and its O-desmethyl metabolite, 12-hydroxyibogamine (noribogaine), in rats. J Pharmacol Exp Ther. 2001;297(2):531-9. |
| --- | --- | --- |
| Cappendijk | 1993 | [^5^](#_ENREF_5) Cappendijk SL, Dzoljic MR. Inhibitory effects of ibogaine on cocaine self-administration in rats. European journal of pharmacology. 1993;241(2-3):261-5. |
| Chen | 1996 | [^6^](#_ENREF_6) Chen K, Kokate TG, Donevan SD, Carroll FI, Rogawski MA. Ibogaine block of the NMDA receptor: in vitro and in vivo studies. Neuropharmacology. 1996;35(4):423-31. |
| Dworkin | 1995 | [^7^](#_ENREF_7) Dworkin SI, Gleeson S, Meloni D, Koves TR, Martin TJ. Effects of ibogaine on responding maintained by food, cocaine and heroin reinforcement in rats. Psychopharmacology (Berl). 1995;117(3):257-61. |
| Geter-Douglass | 1999 | [^8^](#_ENREF_8) Geter-Douglass B, Witkin JM. Behavioral effects and anticonvulsant efficacies of low-affinity, uncompetitive NMDA antagonists in mice. Psychopharmacology (Berl). 1999;146(3):280-9. |
| Glick | 1991 | [^9^](#_ENREF_9) Glick SD, Rossman K, Steindorf S, Maisonneuve IM, Carlson JN. Effects and aftereffects of ibogaine on morphine self-administration in rats. European journal of pharmacology. 1991;195(3):341-5. |
| Glick | 1994 | [^10^](#_ENREF_10) Glick SD, Kuehne ME, Raucci J, Wilson TE, Larson D, Keller RW, Jr., et al. Effects of iboga alkaloids on morphine and cocaine self-administration in rats: relationship to tremorigenic effects and to effects on dopamine release in nucleus accumbens and striatum. Brain research. 1994;657(1-2):14-22. |
| Glick | 1997 | [^11^](#_ENREF_11) Glick SD, Maisonneuve IM, Pearl SM. Evidence for roles of kappa-opioid and NMDA receptors in the mechanism of action of ibogaine. Brain research. 1997;749(2):340-3. |
| He | 2005 | [^12^](#_ENREF_12) He DY, McGough NN, Ravindranathan A, Jeanblanc J, Logrip ML, Phamluong K, et al. Glial cell line-derived neurotrophic factor mediates the desirable actions of the anti-addiction drug ibogaine against alcohol consumption. The Journal of neuroscience : the official journal of the Society for Neuroscience. 2005;25(3):619-28. |
| Helsley | 1997 | [^13^](#_ENREF_13) Helsley S, Dlugos CA, Pentney RJ, Rabin RA, Winter JC. Effects of chronic ibogaine treatment on cerebellar Purkinje cells in the rat. Brain research. 1997;759(2):306-8. |
| Kesner | 1995 | [^14^](#_ENREF_14) Kesner RP, Jackson-Smith P, Henry C, Amann K. Effects of ibogaine on sensory-motor function, activity, and spatial learning in rats. Pharmacology, biochemistry, and behavior. 1995;51(1):103-9. |
| Leal | 2000 | [^15^](#_ENREF_15) Leal MB, de Souza DO, Elisabetsky E. Long-lasting ibogaine protection against NMDA-induced convulsions in mice. Neurochem Res. 2000;25(8):1083-7. |
| Luxton | 1996 | [^16^](#_ENREF_16) Luxton T, Parker LA, Siegel S. Ibogaine fails to interrupt the expression of a previously established one-trial morphine place preference. Progress in neuro-psychopharmacology & biological psychiatry. 1996;20(5):857-72. |
| Molinari | 1996 | [^17^](#_ENREF_17) Molinari HH, Maisonneuve IM, Glick SD. Ibogaine neurotoxicity: a re-evaluation. Brain research. 1996;737(1-2):255-62. |
| Moroz | 1997 | [^18^](#_ENREF_18) Moroz I, Parker LA, Siegel S. Ibogaine interferes with the establishment of amphetamine place preference learning. Exp Clin Psychopharmacol. 1997;5(2):119-22. |
| O'Callaghan | 1996 | [^19^](#_ENREF_19) O'Callaghan JP, Rogers TS, Rodman LE, Page JG. Acute and chronic administration of ibogaine to the rat results in astrogliosis that is not confined to the cerebellar vermis. Ann N Y Acad Sci. 1996;801:205-16. |
| O'Hearn | 1993a | [^20^](#_ENREF_20) O'Hearn E, Molliver ME. Degeneration of Purkinje cells in parasagittal zones of the cerebellar vermis after treatment with ibogaine or harmaline. Neuroscience. 1993;55(2):303-10. |
| O'Hearn | 1993b | [^21^](#_ENREF_21) O'Hearn E, Long DB, Molliver ME. Ibogaine induces glial activation in parasagittal zones of the cerebellum. Neuroreport. 1993;4(3):299-302. |
| O'Hearn | 1995 | [^22^](#_ENREF_22) O'Hearn E, Zhang P, Molliver ME. Excitotoxic insult due to ibogaine leads to delayed induction of neuronal NOS in Purkinje cells. Neuroreport. 1995;6(12):1611-6. |
| O'Hearn | 1997 | [^23^](#_ENREF_23) O'Hearn E, Molliver ME. The olivocerebellar projection mediates ibogaine-induced degeneration of Purkinje cells: a model of indirect, trans-synaptic excitotoxicity. The Journal of neuroscience : the official journal of the Society for Neuroscience. 1997;17(22):8828-41. |
| O'Hearn | 2004 | [^24^](#_ENREF_24) O'Hearn E, Molliver ME. Administration of a non-NMDA antagonist, GYKI 52466, increases excitotoxic Purkinje cell degeneration caused by ibogaine. Neuroscience. 2004;127(2):373-83. |
| Parker | 1995 | [^25^](#_ENREF_25) Parker LA, Siegel S, Luxton T. Ibogaine attenuates morphine-induced conditioned place preference. Exp Clin Psychopharmacol. 1995;3(4):344-8. |
| Pearl | 1996 | [^26^](#_ENREF_26) Pearl SM, Maisonneuve IM, Glick SD. Prior morphine exposure enhances ibogaine antagonism of morphine-induced dopamine release in rats. Neuropharmacology 1996; 35(12): 1779-1784. |
| Rezvani | 1995 | [^27^](#_ENREF_27) Rezvani AH, Overstreet DH, Lee YW. Attenuation of alcohol intake by ibogaine in three strains of alcohol-preferring rats. Pharmacology, biochemistry, and behavior. 1995;52(3):615-20. |
| Scallet | 1996 | [^28^](#_ENREF_28) Scallet AC, Ye X, Rountree R, Nony P, Ali SF. Ibogaine produces neurodegeneration in rat, but not mouse, cerebellum. Neurohistological biomarkers of Purkinje cell loss. Ann N Y Acad Sci. 1996;801:217-26. |
| Sershen | 1994 | [^29^](#_ENREF_29) Sershen H, Hashim A, Lajtha A. Ibogaine reduces preference for cocaine consumption in C57BL/6By mice. Pharmacology, biochemistry, and behavior. 1994;47(1):13-9. |
| Szumlinski | 2000 | [^30^](#_ENREF_30) Szumlinski KK, Maisonneuve IM, Glick SD. Differential effects of ibogaine on behavioural and dopamine sensitization to cocaine. European journal of pharmacology. 2000;398(2):259-62. |
| Trouvin | 1987 | [^31^](#_ENREF_31) Trouvin JH, Jacqmin P, Rouch C, Lesne M, Jacquot C. Benzodiazepine receptors are involved in tabernanthine-induced tremor: in vitro and in vivo evidence. European journal of pharmacology. 1987;140(3):303-9. |
| Xu | 2000 | [^32^](#_ENREF_32) Xu Z, Chang LW, Slikker W, Jr., Ali SF, Rountree RL, Scallet AC. A dose-response study of ibogaine-induced neuropathology in the rat cerebellum. Toxicol Sci. 2000;57(1):95-101. |
| Zetler | 1983 | [^33^](#_ENREF_33) Zetler G. Cholecystokinin octapeptide (CCK-8), ceruletide and analogues of ceruletide: effects on tremors induced by oxotremorine, harmine and ibogaine. A comparison with prolyl-leucylglycine amide (MIF), anti-Parkinsonian drugs and clonazepam. Neuropharmacology. 1983;22(6):757-66. |

| *Table 3: Characteristics table of the included studies (n.exp.ctrl=number of animals in experimental and control group; SUD=substance use disorder; MA=meta-analyse; na=not applicable; i.v.=intravenous; i.p.=intraperitoneal; s.c.=subcutaneous; nr= not reported)* | | | | | | | | | | | | | | | | |
| --- | --- | --- | --- | --- | --- | --- | --- | --- | --- | --- | --- | --- | --- | --- | --- | --- |
| Author | Year | Journal | Species and strain | gender | weight | age | n exp. ctrl | used animal SUD model | type of drug used | Ibogaine dosage | treatment duration | Route of administration | Time window of measurements | Outcome measurement type | N experiments in MA | number of outcomes measures used in MA |
| Bau-mann | 2001 | J Pharm Exper Therapeutics | Rats, Sprague-Dawley | M | 350-400 g | adult | 8, 8 | na | na | 1 mg/kg or 10 mg/kg | single dose | i.v. | + 2,10, 20, 30 min | Motor impairment: tremors | 2 | 2 |
| Cappen dijk | 1993 | Eur J of Pharmacology | Rats, Wistar | M | 200-250 g | nr | 5-8, 5-8 | self-administration | cocaine | 10, 20 and 40 mg/kg | single dose and multiple doses | i.p. | + 2 hours to weeks | Self-administration of cocaine | 2 | 20 |
| Chen | 1996 | Neuropharmacology | Mice , NIH-Swiss | M | 25-30 g | nr | 8-16, ?  Data not obtainable | na | na | 45-55-75-100-120 mg/kg | Single dose | i.p, | + 15 min | Motor impairment : tiled platform test | na | na |
| Dworkin | 1995 | Psychopharmacology | Rats, Fischer 344 | M | 250-300 g | nr | 7-7 for cocaine, 5-5 for heroin | self-administration | cocaine and heroin | 40, 80 mg/kg | single dose | i.p. | + 60 min to 24 h | Self-administration of cocaine or heroin | 2 | 4 |
| Geter-Douglass | 1999 | Psychopharmacology | Mice, Swiss-Webster | M | approx. 30 g | nr | 6-8, 6-8 | na | na | 17, 30, 56 and 100 mg/kg | single dose | s.c. | + 30 minutes | Motor impairment : inverted screen test | 4 | 4 |
| Glick | 1991 | Eur J of Pharmacology | Rats, Sprague-Dawley | F | 230-250 g | approx. 3 months | 4-12, 4-12 | self-administration | morphine | 10, 20, 40, 80 mg/kg | single dose | i.p. | + 15 min - 7 days | Self-administration of morphine | 6 | 14 |
| Glick | 1994 | Brain Research | Rats, Sprague-Dawley | F | 230-250 g | approx. 3 months | 4-8, 4-8 | self-administration | morphine and cocaine | 10,20,30,40 mg/kg | single dose | i.p. | + 15 min - 7 days | Self-administration of morphine, | 2 | 10 |
| Glick | 1994 | Brain Research | Rats, Sprague-Dawley | F | 230-250 g | approx. 3 months | 4-8, 4-8 | na | morphine and cocaine | 10,20,30,40 mg/kg | single dose | i.p. | + 15 min - 4 hours | Motor impairment : tremors | na | na |
| Glick | 1997 | Brain Research | Rats, Sprague-Dawley | F | 230-250 g | approx. 3 months | 6, 6 | self-administration | morphine | 40 mg/kg | single dose | i.p. | + 15 min - 7 days | Self-administration of morphine | 1 | 5 |
| He | 2005 | The J of Neuroscience | Rats: Long Evans | M | nr | adult | 8,8 | self-administration | alcohol | 20-40 mg i.p | single dose | i.p. | + 3 hours | self-administration of alcohol | 3 | 3 |
| He | 2005 | The J of Neuroscience | Mice: C57BL6 | M | nr | adult | 2,2 | na | na | 40 mg i.p | single dose | i.p. | + 12 hours | cerebellar cell loss | 1 | 1 |
| Helsley | 1997 | Brain Research | Rats, Fischer 344 | M | nr | approx. 60 days | 6, 6 | na | na | 10 mg/kg | multiple doses | i.p. | + 48 hours | cerebellar cell loss | 1 | 1 |
| Kesner | 1995 | Pharmac. Biocem and Behaviour | Rats, Long Evans/C57BL/6 | M | nr | approx. 100 days | 8, 2 | na | na | 10,20,30,40,50,60 mg /kg | multiple doses | i.p. | + 30 minutes | Motor impairment : tiled platform test | 6 | 6 |
| Leal | 2000 | Neurochemical Research | Mice , CF-1 | M | 25-35 g | nr | 8, 8 | na | na | 60, 80 mg/kg | single dose | i.p. | + 30 minutes up to72 h | Motor impairment : inverted screen test | 2 | 14 |
| Luxton | 1996 | Prog Neuro-Psychopharmacol & biol psychiat | Rats, Sprague-Dawley | M | 200-224 g | nr | 12-28, 11-28 | place preference | morphine | 40 and 80 mg/kg | single dose and multiple doses | i.p. | + 4 tp 24 hours | Conditioned place preference | 6 | 6 |
| Moliniari | 1996 | Brain Research | Rats, Sprague-Dawley | F | 250-275 g | nr | 2-10, 2 | na | na | 40/100 mg/kg | single dose and multiple doses | i.p. | + 7 days | cerebellar cell loss | 3 | 3 |
| Moroz | 1997 | Exp & clin Psychopharmacology | Rats, Sprague-Dawley | M | 275-305 g | nr | 12-15, 11-16 | place preference | amphetamine | 40 mg/kg | single dose and multiple doses | i.p. | + 48 hours | Conditioned place preference | 4 | 4 |
| O'Callaghan | 1996 | Ann New York Acad sience | Rats, Sprague-Dawley | M+F | nr | 6 weeks | 5, 5 | na | na | 10, 50, 100, 150 mg/kg | single dose and multiple doses | i.p. and p.o. | + 24 hours up until 3 weeks | cerebellar cell loss | 10 | 22 |
| O'Hearn | 1993a | Neuroscience | Rats, Sprague-Dawley | M | 175-200 g | nr | 50, nr  Data not obtainable | na | na | 100 - 300 mg/kg | single dose | i.p. | + 2-15 days | cerebellar cell loss | na | na |
| O'Hearn | 1993b | NeuroReport | Rats, Sprague-Dawley | M | 175-200g | nr | 35, 15 | na | na | 100 - 300 mg/kg | single dose and multiple doses | i.p. | + 1, 2 or 4 weeks | cerebellar cell loss | 1 | 1 |
| O'Hearn | 1995 | NeuroReport | Rats, Sprague-Dawley | M | nr | adult | 3-44, 5 | na | na | 50, 75, 100 mg/kg | single dose and multiple doses three doses each day one dose | i.p. | + 12 hours to 90 days | cerebellar cell loss | 3 | 3 |
| O'Hearn | 1997 | The J of Neuroscience | Rats, Sprague-Dawley | M | 175-220 g | nr | 6, 6 | na | na | 100 mg/kg | single dose and multiple doses | i.p. | + 7 days | cerebellar cell loss | 1 | 1 |
| O'Hearn | 2004 | Neuroscience | Rats, Sprague-Dawley | M | 175-220 g | nr | 15- 20, 3 | na | na | 75, 100 mg/kg | single dose | i.p. | + 5-10 days | cerebellar cell loss | 2 | 2 |
| Parker | 1995 | Exp and Clin Psychopharmacology | Rats, Sprague-Dawley | M | 200-224 g | nr | 7-10, 7-9 | place preference | morphine | 40 mg/kg | single dose and multiple doses | i.p. | + 48 hours | Conditioned place preference | 4 | 4 |
| Pearl | 1996 | Neuropharmacology | Rats, Sprague-Dawley | F | 250-275 g | nr | 4-8, 4-8 | Forced drug intake | Morphine | 10 mg/kg | Single dose | i.p. | 19 hours | Dopamine dialyse leveles | 1 | 1 |
| Rezvani | 1995 | Pharm, Biochem and Behavior | Rats, Fawn Hooded, alcohol preferring and alcohol accepting | M | 460, 550, 470 gr +- 20 g | adult | 8, 3-8 | self-administration | alcohol | 10, 30, 60 mg/kg | single dose | i.p. and intragastric | + 0-24 hour | Self-administration of alcohol | 5 | 14 |
| Scallet | 1996 | Ann New York Acad Science | Mice: C57BL6, Rats: Charles River | M | nr | Mice: 5 month, Rats: 10 month | 7, 6 | na | na | 100 mg/kg | single dose | i.p. | + 7 days | cerebellar cell loss | 2 | 4 |
| Sershen | 1994 | Pharm Biol and Behavior | Mice , C57BL6 | M | nr | 2-4 month | 16, 16 | self-administration | cocaine | 2 times 40 mg/kg 6 hours apart | multiple doses | i.p. | + 0-120 hours | Self-adminstration of cocaine | 1 | 5 |
| Szumlinski | 2000 | Eur J of Pharmacology | Rats, Sprague Dawley | F | nr | nr | 6-8, 6-8 | forced drug intake | cocaine | 40 mg/kg | single dose | i.p. | + 19 hours | dopamine dialysate levels | 1 | 1 |
| Trouvin | 1987 | Eur J of Pharmacology | Rats, Sprague Dawley | M | 200-220 g | nr | nr,  Data not obtainable | na | na | 20 mg/kg | single dose | i.p. | + 5-120 minutes | Motor impairment : tremors | na | na |
| Xu | 2000 | Toxicological Sciences | Rats, Sprague Dawley | F | 319 +_ 3.8 g | 6 month | 6, 6 | na | na | 25,50,75, 100 mg/kg | single dose | i.p. | + 7 days | cerebellar cell loss | 3 | 3 |
| Zetler | 1983 | Neuropharmacology | Mice , NMRI | M | 25 g | nr | 6-10, 10 | na | na | 10 and 20 mg/kg | single dose | s.c. | + 5- 30 min | Motor impairment : tremors | 1 | 2 |

***Table 4****. Articles which studied in vivo neuropharmacological effects of ibogaine on forced drug intake but were not included in the MA.*

| author | journal | species and strain | gender | weight | age | nr of animals experimental and control group | type of drug used | dosing treatment ibogaine | data collection relative to ibogaine treatment | outcome measure type | brain area | results |
| --- | --- | --- | --- | --- | --- | --- | --- | --- | --- | --- | --- | --- |
| Alburges  1999  [^34^](#_ENREF_34) | Brain Research | Rats, Sprague-Dawley | M | 180-230 g | nr | 7,6 | cocaine, single dose 30 mg/kg i.p. | 4x40mg/kg i.p. | 36 hours | neurotensin like immunoreactivity | striatum, Nucleus accumbnes (Nac), Frontal cortex, substantia nigra | ibogaine blocks cocaine induces rising of neurotensin in striatum |
| Alburges  1999b  [^35^](#_ENREF_35) | Brain Research | Rats, Sprague-Dawley | M | 180-230 g | nr | 10,1 | cocaine, single dose 40 mg/kg i.p. | 4x40mg/kg i.p. | 36 hours | dynorphin a like immunoreactivity | striatum, Nac, Frontal cortex | ibogaine enhances cocaine induced rising dynorphin A levels in striatum, Nac and subst nigra |
| Binienda  2000  [^36^](#_ENREF_36) | Ann New York Acad sience | Rats, Sprague-Dawley | M | nr | 3 month | nr | cocaine, single dose 20 mg/kg i.p. | 1x50 mg/kg i.p. | 1 hour | dopamine and serotonin | nucleus caudatus | ibogaine reduces cocaine induced rising dopamine concentrations in nucleus caudatus |
| Broderick  1994  [^37^](#_ENREF_37) | Pharm, Biochem and Behavior | Rats, Sprague-Dawley | M | 327-394 gr | nr | 5,4 | cocaine, single dose 20 mg/kg s.c. | 4x40mg/kg i.p. | 2 hours | dopamine and serotonin release | NAc | ibogaine reduces cocaine induced dopamine concentration and even further reduces cocaine induced decrease of serotonine levels in NAc |
| French  1996  [^38^](#_ENREF_38) | Pharmacology letters | Rats, Sprague-Dawley | nr | 250-350 gr | adult | 6,12 | cocaine 4 mg/kg and morphine 4 mg/kg, single dose | 1x40mg/kg i.p. | 6-8 hours | dopamine induced spiking | Ventral tegmental area (VTA) | ibogaine does not influence cocaine or morphine induced dopamine spiking in VTA |
| Glick  1992  [^39^](#_ENREF_39) | Brain Research | Rats, Sprague-Dawley | F | 250-300 gr | nr | 6,12 | morphine and amphetamine | 1x40mg/kg i.p. | 19 hours | morphine and amphetamine brain levels | whole brain | ibogaine enhances amphetamine brain levels 4 folds, but does not change morphine brain levels |
| Glick  1993  [^40^](#_ENREF_40) | Brain Research | Rats, Sprague-Dawley | F | nr | nr | 5,6 | amphetamine 1,25 mg/kg single dose | 10 mcM cerebral microdialysis 40 minutes | 3 hours | dopamine extracellular levels | striatum, Nac | ibogaine enhances amphetamine-induced dopamine extracellular levels in Nac, but not striatum |
| Levant 2004  [***^41^***](#_ENREF_41) | Brain Research | Rats, Sprague-Dawley | M | 200-220 gr | adult | 10,9 | Morphine 25 mg/kg for 4-7 days | 1x40mg/kg i.p. | 60 minutes | Local cerebral glucose utilization | striatum, Nucleus accumbnes (Nac), Frontal cortex, substantia nigra | Ibogaine produces a global decrease in cerebral glucose utilization in morphine dependent rats with 16 percent |
| Maisonneuve  1991  [^42^](#_ENREF_42) | Eur j Phamracology | Rats, Sprague-Dawley | F | nr | nr | 6,6 | morphine 5 mg/kg i.p., single dose | 1x40 mg/kg i.p,. | 19 hours | dopamine extracellular levels | Nac, striatum, medial prefrontal cortex | ibogaine pre-treatment reduces the rise of morphine induced dopamine in Nac , medial prefrontal cortex and striatum |
| Maisonneuve  1992  [^43^](#_ENREF_43) | Eur j Phamracology | Rats, Sprague-Dawley | M | nr | nr | 6,6 | cocaine 20mg/kg i.p., single dose | 1x40 mg/kg i.p,. | 19 hours | dopamine extracellular levels | Nac, striatum | ibogaine pre-treatment potentiates the rise of cocaine induced dopamine in Nac and striatum |
| Maisonneuve  1992b  [^44^](#_ENREF_44) | Brain Research | Rats, Sprague-Dawley | F | nr | nr | 6,6 | amphetamine 1,25 mg/kg single dose | 1x40 mg/kg i.p,. | 19 hours | dopamine extracellular levels | Nac, striatum | ibogaine pre-treatment potentiates the rise of amphetamine induced dopamine in Nac and striatum |
| Maisonneuve  1992c  [^45^](#_ENREF_45) | Brain Research | Rats, Sprague-Dawley | F | 250-275 gr | nr | 6,6 | morphine 5 mg/kg i.p., single dose | 1x40 mg/kg i.p,. | 19 hours | dopamine post mortem tissue levels | Nac, striatum, prefrontal cortex | ibogaine pre-treatment reduces the rise of morphine induced dopamine in Nac , prefrontal cortex and striatum with about 50% |
| Maisonneuve  1997  [^46^](#_ENREF_46) | Psychopharmacology | Rats, Sprague-Dawley | M | nr | nr | 6,6 | nicotine 0,32 mg/kg i.v., double dose, separated 60 minutes | 1x40 mg/kg i.p,. | 19 hours | dopamine extracellular levels | whole brain | ibogaine pre-treatment reduces dopamine response after nicotine infusion |
| Reid  1996  [^47^](#_ENREF_47) | J Neural Transmission | Rats, Sprague-Dawley | M | nr | nr | 5,5 | cocaine 15 mg/kg i.p. and 10-6M intracerebral, single dose | 5 mcM cerebral micro dialysis 50 minutes | 0-60 minutes | dopamine extracellular levels | Nac, striatum | cocaine pre-treatment does not alter ibogaine induced dopamine increase in striatum and Nac |
| Sershen  1992  [^48^](#_ENREF_48) | Life Sciences | Mice, C57BL/6By | M | nr | 2-4 month | 8,8 | amphetamine 5 mg/kg single dose s.c. | 2x40 mg/kg s.c. | 3 hours | dopamine post mortem tissue levels | striatum | After ibogaine pre-treatment amphetamine induced striatal dopamine release is not altered |

***Table 5: Effects of ibogaine on drug induced conditioned place preference in different subgroups for all comparisons (n=12). (Low dose: 0-40 mg/kg; Medium dose: 40-80 mg/kg; High dose: >80 mg/kg). None of the subgroups showed a significant difference.***

| ***Subgroup*** | ***SMD*** | ***Lower limit*** | ***Upper limit*** | ***n*** | ***I^2^ %*** |
| --- | --- | --- | --- | --- | --- |
| Overall | -0.22 | -0.53 | 0.08 | 14 | 39 |
| 0-24 hours | -0.01 | -0.40 | 0.37 | 5 | 0 |
| > 24 hours | -0.38 | -0.83 | 0.08 | 9 | 56 |
| Amphetamine | -0.38 | -0.83 | 0.47 | 4 | 73 |
| Opioids | 0.03 | -0.47 | 0.53 | 10 | 61 |
| Low dose | -0.45 | -0.99 | 0.10 | 6 | 46 |
| Medium dose | -0.18 | -0.59 | 0.23 | 4 | 14 |
| High dose | 0.04 | -0.62 | 0.69 | 4 | 48 |
| 1 CPP cycle | -0.26 | -0.62 | 0.10 | 9 | 34 |
| >1 CPP cycle | -0.16 | -0.78 | 0.46 | 5 | 56 |

***Table 6: Effects of ibogaine on motor functioning in different subgroups for continuous measurements (blue, n=10) and for dichotomous measurements (green, n=6). (Low dose: 0-40 mg/kg; Medium dose: 40-80 mg/kg; High dose: >80 mg/kg)). None of the subgroups showed a significant difference. Subgroup analyses were conducted when the subgroups contained at least 4 comparisons.***

| ***Subgroup*** | ***SMD*** | ***Lower limit*** | ***Upper limit*** | ***n*** | ***I^2^ %*** |
| --- | --- | --- | --- | --- | --- |
| Overall continuous | 0.82 | 0.46 | 1.117 | 10 | 0 |
| Low dose | 1.11 | 0.44 | 1.78 | 5 | 6 |
| Medium dose | 0.61 | 0.07 | 1.15 | 4 | 0 |
| High dose |  |  |  | 1 |  |
| Mice | 0.66 | 0.19 | 1.14 | 2 | 0 |
| Rat | 1.02 | 0.48 | 1.56 | 8 | 0 |
| ***Subgroup*** | ***RR*** | ***Lower limit*** | ***Upper limit*** | ***n*** | ***I^2^ %*** |
| Overall dichotomous | 6.20 | 2.20 | 17.44 | 6 | 15 |
| Low dose | 5.61 | 1.15 | 27.42 | 4 | 46 |
| Medium dose |  |  |  | 1 |  |
| High dose |  |  |  | 1 |  |

***Table 7: Effects of ibogaine on cerebral cell loss in different subgroups for comparisons with continuous measurements (blue, n=13) and for dichotomous measurements (green, n=15). (Low dose: 0-40 mg/kg (not present in continuous measurements); Medium dose: 40-80 mg/kg; High dose: >80 mg/kg; *=p<0.05). Subgroup analyses were conducted when the subgroups contained at least 4 comparisons.***

| ***Subgroup*** | ***SMD*** | ***Lower limit*** | ***Upper limit*** | ***n*** | ***I^2^ %*** |
| --- | --- | --- | --- | --- | --- |
| Overall continuous | 0.78 | 0.32 | 1.23 | 13 | 42 |
| Intraperitoneal | 1.27 | 0.87 | 1.66 | 7 | 45 |
| Per os | -0.22 | -0.89 | 0.45 | 6 | 47 * |
| Medium dose |  |  |  | 2 |  |
| High dose | 0.63 | 0.12 | 1.15 | 11 | 46 |
| High dose (no per os dosing) | 1.21 | 0.68 | 1.73 | 5 | 33 |
| Female | 0.78 | 0.01 | 1.56 | 5 | 45 |
| Male | 0.75 | 0.14 | 1.36 | 8 | 47 |
| Female (no per os dosing) | 1.48 | 0.82 | 2.14 | 7 | 0 |
| Male (no per os dosing) | 1.16 | 0.59 | 1.72 | 8 | 25 |
| 0-24 hours after ibogaine dosing | -0.22 | -0.89 | 0.45 | 6 | 0 * (as compared to the >72 hours group) |
| 24-72 hours |  |  |  | 1 |  |
| >72 hours | 1.41 | 0.99 | 1.82 | 6 | 0 |
| ***Subgroup*** | ***RR*** | ***Lower limit*** | ***Upper limit*** | ***n*** | ***I^2^ %*** |
| Overall dichotomous | 2.60 | 1.35 | 5.01 | 15 | 0 |
| Low dose |  |  |  | 3 |  |
| Medium dose | 2.91 | 0.85 | 9.96 | 4 | 0 |
| High dose | 3.89 | 1.64 | 9.23 | 8 | 0 |
| Mice |  |  |  | 2 |  |
| Rats | 2.84 | 1.44 | 5.59 | 13 | 0 |
| Female | 1.48 | 0.60 | 3.63 | 7 | 0 |
| Male | 4.95 | 1.89 | 12.92 | 8 | 0 |
| 0-24 hours |  |  |  | 1 |  |
| >72 hours | 2.69 | 1.38 | 5.23 | 14 | 0 |

***Fig 1****.* *Results of the risk of bias assessment of the 30 studies included in this systematic review. The first two items assess study quality by scoring reporting, a ‘yes’ score indicating reported, and a ‘no’ score indicating unreported. The other items assessed risk of bias, with ‘yes’ indicating low risk of bias, ‘no’ high risk of bias and ‘?’ unclear risk of bias.*


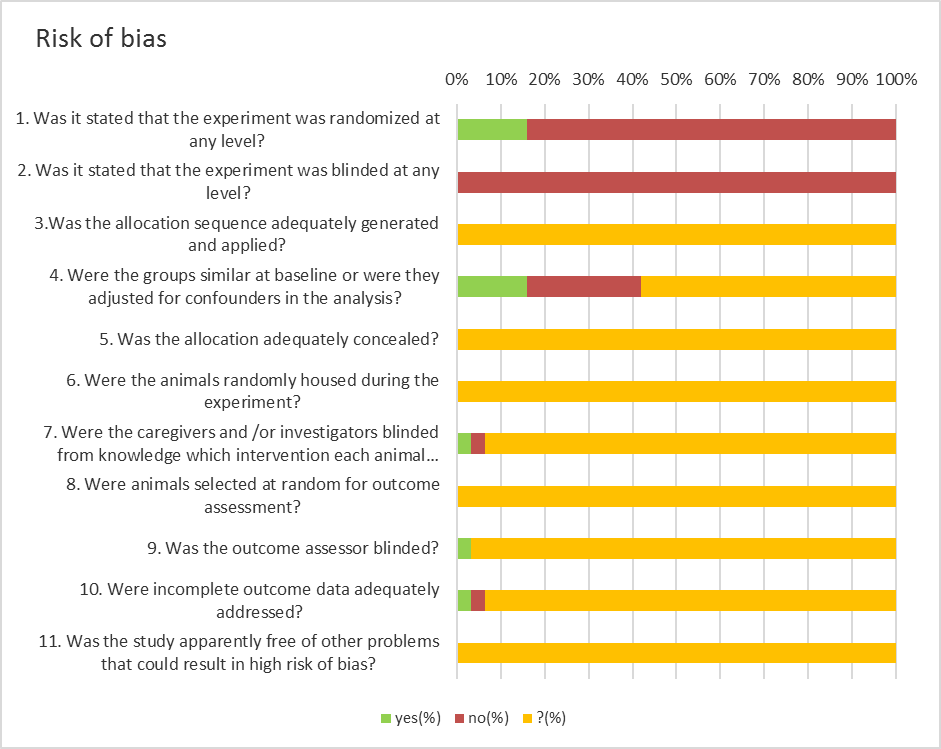


***Fig 2****. Assessment of publication bias in the studies on drug self-administration. Inspection of the funnel plot suggests an underrepresentation of studies with moderate precision and an increased numbers of drug self-administration as a consequence of treatment with ibogaine. Black circles represent the observed data, red circles the estimations of the missing data.*

**-8**

**-7**

**-6**

**-5**

**-4**

**-3**

**-2**

**-1**

**0**

**1**

**2**

**3**

**4**

**5**

**6**

**7**

**8**

**0**

**1**

**2**

**3**

**Standard Error**

**Std diff in means**

References supplemental material

1. Hooijmans CR, Tillema A, Leenaars M, Ritskes-Hoitinga M. Enhancing search efficiency by means of a search filter for finding all studies on animal experimentation in PubMed. *Laboratory animals* 2010; **44**(3)**:** 170-175.

2. de Vries RB, Hooijmans CR, Tillema A, Leenaars M, Ritskes-Hoitinga M. A search filter for increasing the retrieval of animal studies in Embase. *Lab Anim* 2011; **45**(4)**:** 268-270.

3. de Vries RB, Hooijmans CR, Tillema A, Leenaars M, Ritskes-Hoitinga M. Updated version of the Embase search filter for animal studies. *Lab Anim* 2014; **48**(1)**:** 88.

4. Baumann MH, Rothman RB, Pablo JP, Mash DC. In vivo neurobiological effects of ibogaine and its O-desmethyl metabolite, 12-hydroxyibogamine (noribogaine), in rats. *J Pharmacol Exp Ther* 2001; **297**(2)**:** 531-539.

5. Cappendijk SL, Dzoljic MR. Inhibitory effects of ibogaine on cocaine self-administration in rats. *European journal of pharmacology* 1993; **241**(2-3)**:** 261-265.

6. Chen K, Kokate TG, Donevan SD, Carroll FI, Rogawski MA. Ibogaine block of the NMDA receptor: in vitro and in vivo studies. *Neuropharmacology* 1996; **35**(4)**:** 423-431.

7. Dworkin SI, Gleeson S, Meloni D, Koves TR, Martin TJ. Effects of ibogaine on responding maintained by food, cocaine and heroin reinforcement in rats. *Psychopharmacology* 1995; **117**(3)**:** 257-261.

8. Geter-Douglass B, Witkin JM. Behavioral effects and anticonvulsant efficacies of low-affinity, uncompetitive NMDA antagonists in mice. *Psychopharmacology* 1999; **146**(3)**:** 280-289.

9. Glick SD, Rossman K, Steindorf S, Maisonneuve IM, Carlson JN. Effects and aftereffects of ibogaine on morphine self-administration in rats. *European journal of pharmacology* 1991; **195**(3)**:** 341-345.

10. Glick SD, Kuehne ME, Raucci J, Wilson TE, Larson D, Keller RW, Jr.*, et al*. Effects of iboga alkaloids on morphine and cocaine self-administration in rats: relationship to tremorigenic effects and to effects on dopamine release in nucleus accumbens and striatum. *Brain research* 1994; **657**(1-2)**:** 14-22.

11. Glick SD, Maisonneuve IM, Pearl SM. Evidence for roles of kappa-opioid and NMDA receptors in the mechanism of action of ibogaine. *Brain research* 1997; **749**(2)**:** 340-343.

12. He DY, McGough NN, Ravindranathan A, Jeanblanc J, Logrip ML, Phamluong K*, et al*. Glial cell line-derived neurotrophic factor mediates the desirable actions of the anti-addiction drug ibogaine against alcohol consumption. *The Journal of neuroscience : the official journal of the Society for Neuroscience* 2005; **25**(3)**:** 619-628.

13. Helsley S, Dlugos CA, Pentney RJ, Rabin RA, Winter JC. Effects of chronic ibogaine treatment on cerebellar Purkinje cells in the rat. *Brain research* 1997; **759**(2)**:** 306-308.

14. Kesner RP, Jackson-Smith P, Henry C, Amann K. Effects of ibogaine on sensory-motor function, activity, and spatial learning in rats. *Pharmacology, biochemistry, and behavior* 1995; **51**(1)**:** 103-109.

15. Leal MB, de Souza DO, Elisabetsky E. Long-lasting ibogaine protection against NMDA-induced convulsions in mice. *Neurochem Res* 2000; **25**(8)**:** 1083-1087.

16. Luxton T, Parker LA, Siegel S. Ibogaine fails to interrupt the expression of a previously established one-trial morphine place preference. *Progress in neuro-psychopharmacology & biological psychiatry* 1996; **20**(5)**:** 857-872.

17. Molinari HH, Maisonneuve IM, Glick SD. Ibogaine neurotoxicity: a re-evaluation. *Brain research* 1996; **737**(1-2)**:** 255-262.

18. Moroz I, Parker LA, Siegel S. Ibogaine interferes with the establishment of amphetamine place preference learning. *Exp Clin Psychopharmacol* 1997; **5**(2)**:** 119-122.

19. O'Callaghan JP, Rogers TS, Rodman LE, Page JG. Acute and chronic administration of ibogaine to the rat results in astrogliosis that is not confined to the cerebellar vermis. *Ann N Y Acad Sci* 1996; **801:** 205-216.

20. O'Hearn E, Molliver ME. Degeneration of Purkinje cells in parasagittal zones of the cerebellar vermis after treatment with ibogaine or harmaline. *Neuroscience* 1993; **55**(2)**:** 303-310.

21. O'Hearn E, Long DB, Molliver ME. Ibogaine induces glial activation in parasagittal zones of the cerebellum. *Neuroreport* 1993; **4**(3)**:** 299-302.

22. O'Hearn E, Zhang P, Molliver ME. Excitotoxic insult due to ibogaine leads to delayed induction of neuronal NOS in Purkinje cells. *Neuroreport* 1995; **6**(12)**:** 1611-1616.

23. O'Hearn E, Molliver ME. The olivocerebellar projection mediates ibogaine-induced degeneration of Purkinje cells: a model of indirect, trans-synaptic excitotoxicity. *The Journal of neuroscience : the official journal of the Society for Neuroscience* 1997; **17**(22)**:** 8828-8841.

24. O'Hearn E, Molliver ME. Administration of a non-NMDA antagonist, GYKI 52466, increases excitotoxic Purkinje cell degeneration caused by ibogaine. *Neuroscience* 2004; **127**(2)**:** 373-383.

25. Parker LA, Siegel S, Luxton T. Ibogaine attenuates morphine-induced conditioned place preference. *Exp Clin Psychopharmacol* 1995; **3**(4)**:** 344-348.

26. Pearl SM, Maisonneuve IM, Glick SD. Prior morphine exposure enhances ibogaine antagonism of morphine-induced dopamine release in rats. *Neuropharmacology* 1996; **35**(12)**:** 1779-1784.

27. Rezvani AH, Overstreet DH, Lee YW. Attenuation of alcohol intake by ibogaine in three strains of alcohol-preferring rats. *Pharmacology, biochemistry, and behavior* 1995; **52**(3)**:** 615-620.

28. Scallet AC, Ye X, Rountree R, Nony P, Ali SF. Ibogaine produces neurodegeneration in rat, but not mouse, cerebellum. Neurohistological biomarkers of Purkinje cell loss. *Ann N Y Acad Sci* 1996; **801:** 217-226.

29. Sershen H, Hashim A, Lajtha A. Ibogaine reduces preference for cocaine consumption in C57BL/6By mice. *Pharmacology, biochemistry, and behavior* 1994; **47**(1)**:** 13-19.

30. Szumlinski KK, Maisonneuve IM, Glick SD. Differential effects of ibogaine on behavioural and dopamine sensitization to cocaine. *European journal of pharmacology* 2000; **398**(2)**:** 259-262.

31. Trouvin JH, Jacqmin P, Rouch C, Lesne M, Jacquot C. Benzodiazepine receptors are involved in tabernanthine-induced tremor: in vitro and in vivo evidence. *European journal of pharmacology* 1987; **140**(3)**:** 303-309.

32. Xu Z, Chang LW, Slikker W, Jr., Ali SF, Rountree RL, Scallet AC. A dose-response study of ibogaine-induced neuropathology in the rat cerebellum. *Toxicol Sci* 2000; **57**(1)**:** 95-101.

33. Zetler G. Cholecystokinin octapeptide (CCK-8), ceruletide and analogues of ceruletide: effects on tremors induced by oxotremorine, harmine and ibogaine. A comparison with prolyl-leucylglycine amide (MIF), anti-Parkinsonian drugs and clonazepam. *Neuropharmacology* 1983; **22**(6)**:** 757-766.

34. Alburges ME, Hanson GR. Differential responses by neurotensin systems in extrapyramidal and limbic structures to ibogaine and cocaine. *Brain research* 1999; **818**(1)**:** 96-104.

35. Alburges ME, Hanson GR. Ibogaine pretreatment dramatically enhances the dynorphin response to cocaine. *Brain research* 1999; **847**(1)**:** 139-142.

36. Binienda Z, Beaudoin MA, Thorn BT, Sadovova N, Skinner RD, Slikker W, Jr.*, et al*. Application of electrophysiological method to study interactions between ibogaine and cocaine. *Ann N Y Acad Sci* 2000; **914:** 387-393.

37. Broderick PA, Phelan FT, Eng F, Wechsler RT. Ibogaine modulates cocaine responses which are altered due to environmental habituation: in vivo microvoltammetric and behavioral studies. *Pharmacology, biochemistry, and behavior* 1994; **49**(3)**:** 711-728.

38. French ED, Dillon K, Ali SF. Effects of ibogaine, and cocaine and morphine after ibogaine, on ventral tegmental dopamine neurons. *Life Sci* 1996; **59**(12)**:** PL199-205.

39. Glick SD, Gallagher CA, Hough LB, Rossman KL, Maisonneuve IM. Differential effects of ibogaine pretreatment on brain levels of morphine and (+)-amphetamine. *Brain research* 1992; **588**(1)**:** 173-176.

40. Glick SD, Rossman K, Wang S, Dong N, Keller RW, Jr. Local effects of ibogaine on extracellular levels of dopamine and its metabolites in nucleus accumbens and striatum: interactions with D-amphetamine. *Brain research* 1993; **628**(1-2)**:** 201-208.

41. Levant B, Pazdernik TL. Differential effects of ibogaine on local cerebral glucose utilization in drug-naive and morphine-dependent rats. *Brain research* 2004; **1003**(1-2)**:** 159-167.

42. Maisonneuve IM, Keller RW, Jr., Glick SD. Interactions between ibogaine, a potential anti-addictive agent, and morphine: an in vivo microdialysis study. *European journal of pharmacology* 1991; **199**(1)**:** 35-42.

43. Maisonneuve IM, Glick SD. Interactions between ibogaine and cocaine in rats: in vivo microdialysis and motor behavior. *European journal of pharmacology* 1992; **212**(2-3)**:** 263-266.

44. Maisonneuve IM, Keller RW, Jr., Glick SD. Interactions of ibogaine and D-amphetamine: in vivo microdialysis and motor behavior in rats. *Brain research* 1992; **579**(1)**:** 87-92.

45. Maisonneuve IM, Rossman KL, Keller RW, Jr., Glick SD. Acute and prolonged effects of ibogaine on brain dopamine metabolism and morphine-induced locomotor activity in rats. *Brain research* 1992; **575**(1)**:** 69-73.

46. Maisonneuve IM, Mann GL, Deibel CR, Glick SD. Ibogaine and the dopaminergic response to nicotine. *Psychopharmacology* 1997; **129**(3)**:** 249-256.

47. Reid MS, Hsu K, Jr., Souza KH, Broderick PA, Berger SP. Neuropharmacological characterization of local ibogaine effects on dopamine release. *J Neural Transm* 1996; **103**(8-9)**:** 967-985.

48. Sershen H, Harsing LG, Jr., Hashim A, Lajtha A. Ibogaine reduces amphetamine-induced locomotor stimulation in C57BL/6By mice, but stimulates locomotor activity in rats. *Life Sci* 1992; **51**(13)**:** 1003-1011.
